# Phylogenetic and Morphological Analyses Reveal Twelve New Species of the Genus *Patellaria* (Dothideomycetes, Ascomycota) from Mexico

**DOI:** 10.3390/jof11010044

**Published:** 2025-01-07

**Authors:** Ilian García-Jacobo, Tania Raymundo, Cesar R. Martínez-González, Michelle Martínez-Pineda, Ricardo Valenzuela

**Affiliations:** 1 Laboratorio de Micología, Departamento de Botánica, Escuela Nacional de Ciencias Biológicas, Instituto Politécnico Nacional, Prolongación de Carpio and Plan de Ayala, Santo Tomás, Alcaldía Miguel Hidalgo, Ciudad de Mexico 11340, Mexicommartinezipn@ipn.mx (M.M.-P.); rvalenzg@ipn.mx (R.V.); 2 Departamento de Fitotécnia de México, Instituto de Horticultura, Universidad Autónoma Chapingo, km 38.5 Carretera Federal México-Texcoco, Texcoco 56230, Mexico; ramiro_mg.unam@ciencias.unam.mx

**Keywords:** *Patellaria*, phylogeny, taxonomy, morphology, xerophytic scrub

## Abstract

*Patellaria* species are widely distributed in terrestrial and marine habitats and are saprobes growing on decaying wood, stems, or bark. However, studies on this genus in Mexico are limited, and only the type species *Patellaria atrata* Fr. has been cited. This study describes twelve new *Patellaria* species in Mexico supported by molecular (ITS-LSU-SSU) and morphological data. Phylogenetic analysis shows that species of this genus in Mexico are not closely related to *Patellaria atrata.* Moreover, the results demonstrate that the greatest species diversity is found in dry climates, particularly in xerophilous scrub.

## 1. Introduction

*Patellaria* was described by Fries in 1822 [1] and is characterized by superficial ascomata, black or dark coloration, is sessile, and has a dark green to black epithecium, which is formed by branched and rounded pseudoparaphyses, cylindrical and pedicellate asci, and clavate to allantoid septate ascospores; currently, it is represented by around 50 morphological species [2].

*Patellaria* species are widely distributed in terrestrial and marine habitats [3,4] and are saprobes on decaying wood, stems, or bark. *Patellaria atrata* Fr., as the type species, is the most widely distributed species and has been cited as growing on several woody hosts worldwide [3]. This species has apotecioid ascomata 675–1160 µm long, ascospores 30–45 × 7–10 µm, and 5–11 septa [5].

Most species of this genus have been described based on morphological features, including the shape and color of the ascomata, the shape and size of the asci, and the size and number of septa in the ascospores, and only five species have been described using morphological and molecular data: *Patellaria quercus* Crous & R.K. Schumach. from Germany [6], *P. apiculatae* Dayar. and K.D. Hyde and *P. chromolaenae* Mapook and K.D. Hyde from Thailand [3,7], *P. microspora* Ekanayaka and K.D. Hyde from the United Kingdom [8], and *P. guizhouensis* J.F. Zhang, Y.Y. Chen, and Z.Y. Liu from China [9]. Recent studies on the genus have revealed that *P. chromolaena* demonstrates antibacterial activity against *E. coli* [7]. Therefore, in Mexico, studies on *Patellaria* are scarce, and only *P. atrata* has been cited in Chiapas, Veracruz [10], Sonora [11,12], and Quintana Roo [13,14], and only with morphological support. This study aims to analyze specimens of the genus *Patellaria* collected in Mexico using morphological and molecular characteristics for species delimitation.

## 2. Materials and Methods

### 2.1. Morphological Studies

Specimens deposited in the herbarium of the fungal collection of the Escuela Nacional de Ciencias Biológicas, Instituto Politécnico Nacional (ENCB) were collected from decorticated wood or dead twigs or branches in the tropical dry forest, xerophilous scrub, tropical rain forest, and mangroves between 2014 and 2023; a total of 40 specimens were examined in this study. Macroscopic characteristics, such as the size and color of the ascomata, were measured using a stereomicroscope (Leica S9E, Leica, Wetzlar, Germany), and microscopical observations were made of cross-sections from the middle part of the ascomata; mounted in temporary slides; and rehydrated in 70% alcohol, 5% KOH, and water. The microscopical characters were described according to Yacharoen et al. [5], and the peridium, epithecium, asci, pseudoparaphyses, and ascospores were measured and characterized using an optical microscope (Olympus CX31, Olympus, Tokyo, Japan). The color key for the epithecium was determined using the *Methuen Handbook of Colour* [15].

### 2.2. Amplification and Sequencing

We obtained DNA from herbarium specimens. Genomic DNA was extracted using the CTAB method [16]. The DNA was quantified with a Nanodrop 2000c (Thermo, Waltham, MA, USA). We prepared dilutions from each sample at 20 ng/µL to amplify the next regions: the Internal Transcribed Spacer rDNA-ITS1 5.8S rDNA-ITS2 (ITS), the nuclear large subunit ribosomal DNA (nLSU), and the region of the mitochondrial small subunit (mtSSU) (Table 1). The reaction mixture for PCRs was used with a final volume of 15 µL containing 1× buffer, 0.8 mM of dNTPs mix, 20 pmol of each primer, 2 units of GoTaq DNA (Promega, Madison, WI, USA), and 100 ng of template DNA. The PCR products were verified by agarose gel electrophoresis. The gels were run for 1 h at 95 V cm^−3^ in 1.5% agarose and 1× TAE buffer (Tris Acetate-EDTA). The gel was stained with GelRed (Biotium, Fremont, CA, USA), and the bands were visualized in an Infinity 3000 transilluminator (Vilber Lourmat, Eberhardzell, Germany). The amplified products were purified with the ExoSAP Purification kit (Affymetrix, Santa Clara, CA, USA), following the manufacturer’s instructions. They were quantified and prepared for the sequence reaction using a BigDye Terminator v.3.1 (Applied Biosystems, Waltham, MA, USA). These products were sequenced in both directions with an Applied Biosystem model 3730XL (Applied BioSystems, Foster City, CA, USA) at the Instituto de Biología of the Universidad Nacional Autónoma de México (UNAM). The sequences of both strands of each of the genes were analyzed, edited, and assembled using BioEdit v. 7.0.5 [17] to generate a consensus sequence that was compared with those deposited in GenBank (2020) using BLASTN v. 2.2.9 [18].

### 2.3. Phylogenetic Analysis

To explore the phylogenetic relationships of the new species of *Patellaria*, an alignment was made based on the taxonomic sampling employed by Maharachchikumbura et al. [19] (Table 2). Each gene region was independently aligned using the online version of MAFFT v. 7 [20,21,22]. Alignment was reviewed in PhyDE v.10.0 [23], followed by minor manual adjustments to ensure character homology between taxa. The matrix was formed for ITS by 36 taxa (695 characters), nLSU by 42 taxa (850 characters), and SSU by 29 taxa (643 characters). Three partitioning schemes were established: one for the ITS, one for the nLSU, and one SSU, which were established using the option to minimize the stop codon with Mesquite v3.70 [24]. The data were analyzed using maximum parsimony (MP), maximum likelihood (ML), and Bayesian inference (BI). Maximum parsimony analyses were conducted in PAUP* 4.0b10 [25] using the heuristic search mode, 1000 random starting replicates, and TBR branch swapping, with MULTREES and Collapse on. Bootstrap values were estimated using 1000 bootstrap replicates under the heuristic search mode, each with 100 random starting replicates. Maximum likelihood analyses were conducted in RAxML v. 8.2.10 [26] with a GTR + G model of nucleotide substitution. To assess branch support, 10,000 rapid bootstrap replicates were run with the GTRGAMMA model. Bayesian inference was conducted in MrBayes v. 3.2.6 x64 [27] with four chains, and the best evolutionary model for alignment was sought using PartitionFinder [28,29,30]. Phylogenetic analyses were performed using MrBayes v3.2.6 x64 [27]. The information block for the matrix included two simultaneous runs of Montecarlo chains, a temperature set to 0.2, and sampling of 10 million generations (standard deviation ≤ 0.1). Chain convergence was visualized in Tracer v.1.7 [31]. The remaining trees were used to calculate a 50% majority-rule consensus topology and posterior probabilities (PP). Trees were visualized and optimized in FigTree v. 1.1.4 [32], and they were edited in Adobe Illustrator (Adobe Systems, Inc., San Jose, CA, USA).

**Table 1:** The primers used in the amplification and sequencing of the DNA fragments.

Loci/Segment	Primer	Sequence 5′-3′	T (°C)	Reference
ITS	ITS5	GGAAGTAAAAGTCGTAACAAGG	58	[33]
	ITS4	TCCTCCGCTTATTGATATGC	58	[33]
nLSU	LROR	ACCCGCTGAACTTAAGC	48	[33]
	LR3	GGTCCGTGTTTCAAGAC	48	[33]
0mtSSU	MS1	CAGCAGTCAAGAATATTAGTCAATG	63	[33]
	MS2	GCGGATTATCGAATTAAATAAC	53	[33]

## 3. Results

### 3.1. Phylogenetic Analysis

**Figure 1:**
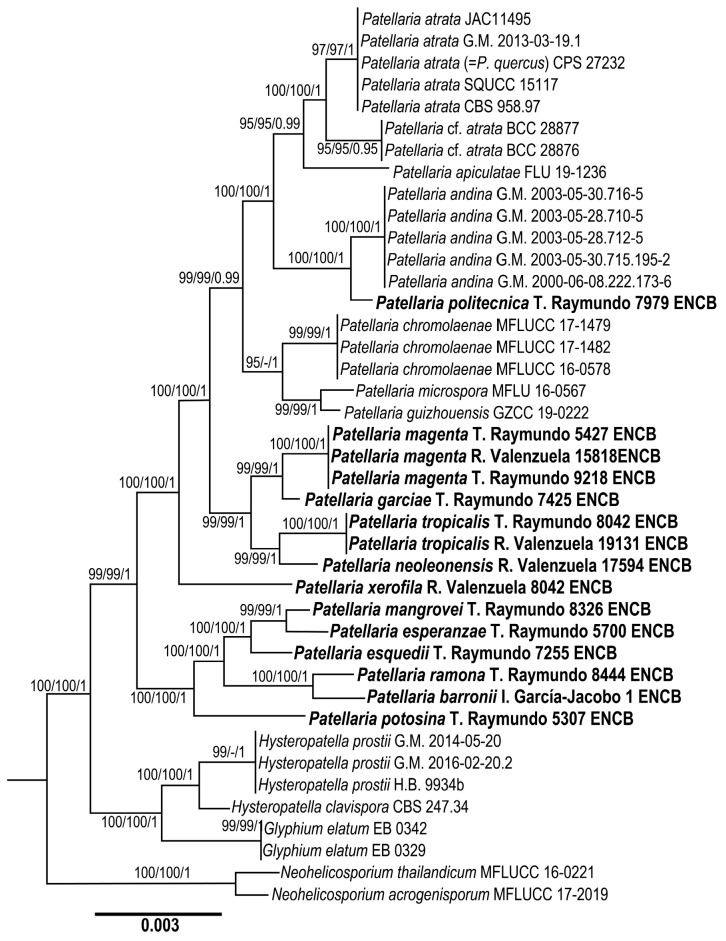
Maximum likelihood phylogeny based on the concatenated ITS, nLSU, and SSU sequence alignment. Maximum parsimony and Bayesian analyses recovered identical topologies with respect to the relationships among the main clades of members of Patellaria. For each node, the following values are provided: the maximum parsimony bootstrap (%)/maximum likelihood bootstrap (%) and posterior confidence (*p*-value). The scale bar represents the expected number of nucleotide substitutions per site.

The dataset of ITS, nLSU, and SSU combined comprises 42 taxa with 2300 characters, including gaps. The three phylogenetic analyses of the dataset, MP, ML, and BI, recovered similar topologies (Figure 1). No significant conflict (bootstrap value > 80%) was detected among the topologies obtained via the separate phylogenetic analyses. The parsimony analysis of the alignment found 942 trees of 112 steps (CI = 0.1002, HI = 0.1004, RI = 0.2062, RC = 0.1197). The best RAxML tree with a final likelihood value of −20624.050971 is presented. The matrix had 542 distinct alignment patterns, with 3.07% undetermined characters or gaps. Estimated base frequencies were as follows: A = 0.108340, C = 0.108312, G = 0.100074, T = 0.100214; substitution rates: AC = 1.000927, AG = 1.008320, AT = 1.000734, CG = 1.000831, CT = 4.000347, GT = 1.100000; gamma distribution shape parameter α = 0.002000. In the Bayesian analysis, the standard deviation between the chains stabilized at 0.002 after 5.2 million generations. No significant changes in tree topology trace or cumulative split frequencies of selected nodes were observed after about 0.25 million generations, which were discarded as 25% burn-in. The analysis produced a phylogenetic tree where *Patellaria* is shown as a monophyletic group (BS = 100%, BS = 100%, BI *p* = 1).

### 3.2. Taxonomy

***Patellaria barronii*** García-Jacobo, Raymundo, and R. Valenz. sp. nov.

**MycoBank**: 855485.

**Figure 2:**
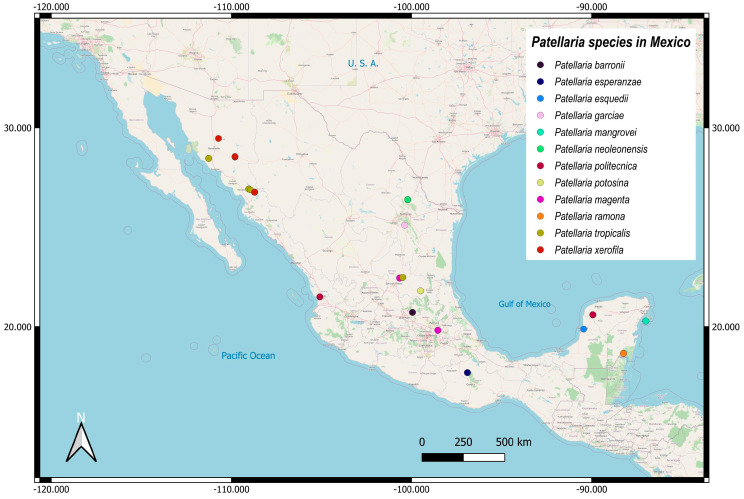
The distribution of new *Patellaria* species.

**Figure 3:**
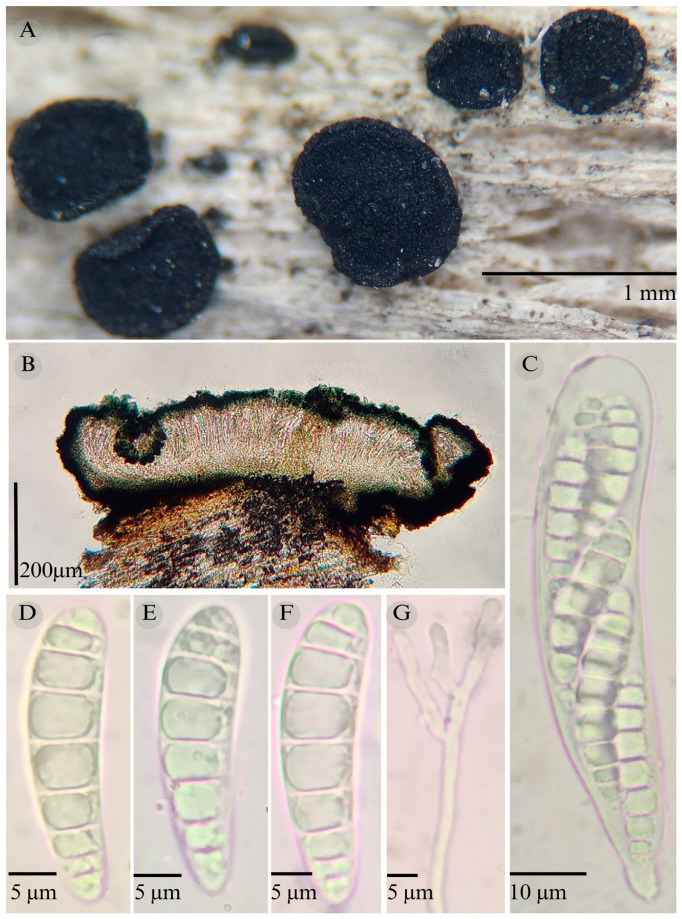
*Patellaria barronii* García-Jacobo, Raymundo, and R. Valenz. (**A**) Ascomata, (**B**) longitudinal section of ascomata, (**C**) asci, (**D**–**F**) ascospores, and (**G**) pseudoparaphysis.

**Figures:** Figure 1, Figure 2 and Figure 3.

**TYPE**: Mexico. Querétaro, Bernal municipality, Cerca del Gallito, Peña de Bernal, 20°44′45.0″ N, 99°56′44.5″ W, 2145 m, 24 December 2023, S. García 1 (ENCB-Holotype).

**Etymology**: In honor of Dr. Isaac Silva Barrón, who supported the study of mycology in Querétaro.

Sexual morph: Ascomata of 500–730 × 500–560 µm, apothecioid, superficial, sessile, circular, flattened, with a thin carbonaceous rim, exposing a black hymenium at the center; peridium 33–40 µm wide ($\bar{x}$ = 37, *n* = 10), composed of two layers, outer layer of 20–22 µm ($\bar{x}$ = 21.2, n = 10), pseudoparenchymatous, black, composed of thick-walled cells 4–6.5 × 4–6 µm wide, inner layer of 15 to 25 µm ($\bar{x}$ = 20, *n* = 10), textura prismatica, composed of thick-walled cells; subhymenium of 35 to 40 µm ($\bar{x}$ = 37, *n* = 3), hyaline to greenish blue, composed of thick-walled cells; hamathecium 1–3 µm wide, thick-walled, filiform to cylindrical, septate, branched, hyaline pseudoparaphyses, rounded at the apex, forming a green jungle (25E7) to black thick epithecium over the asci when mounted in KOH ($\bar{x}$ = 20 µm, *n* = 10); bitunicate asci, 96–101.3 × 14.8–15.3 µm, ($\bar{x}$ = 101.33 × 15.3, *n* = 10), cylindrical, clavate, apically rounded, generally straight, with a short unilobed pedicel, eight-spored, bitunicate; ascospores (28) 31–35 (36) × 6–8 µm, ($\bar{x}$ = 31.46 × 6.33, *n* = 30), clavate, narrowed at the lower end, 6–8 (9) septa, hyaline refractive in a pale green tone. Asexual morph: unknown.

**Notes:** This species is distinguished by the ascospores size (28–36 × 5–8) and number of septa (6–8). Morphologically, a similar species is *Patellaria xerofila*; both grow in the same type of vegetation (xerophilous scrub). However, *P. xerofila* differs by the dark turquoise pigmentation in KOH and ascospores 28–30 × 6–7 µm with septa (5–7). Phylogenetically, it is related to *P. ramona*, but differs by having larger asci ($\bar{x}$ = 101.33 µm vs. $\bar{x}$ = 88.5 µm), larger ascospores ($\bar{x}$ = 31.46 µm vs. $\bar{x}$ = 21.11 µm), and more septa (6–8 vs. 4–6). Therefore, *P. barronii* is described as a new species based on phylogeny and morphology comparison.

***Patellaria esperanzae*** García-Jacobo and Raymundo sp. nov.

**MycoBank**: 855490.

**Figure 4:**
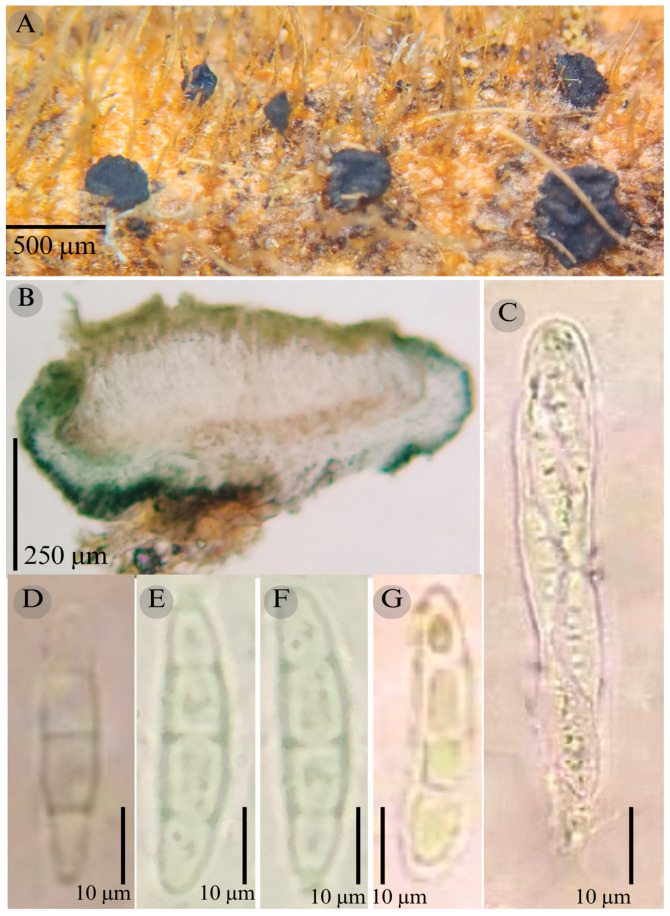
*Patellaria esperanzae* García-Jacobo, and Raymundo. (**A**) Ascomata, (**B**) longitudinal section of ascomata, (**C**) asci, and (**D**–**G**) ascospores.

**Figures:** Figure 1, Figure 2 and Figure 4.

**TYPE**: Mexico. Oaxaca, Dto. Ixtlán de Juárez, Santiago Comaltepec Municipality, La Esperanza, 17°35′28.1″ N, 96°53′52.2″ W, 1398 m, 15 May 2015; T. Raymundo 5700 (ENCB, Holotype)

**Etymology**: The epithet refers to the locality “La Esperanza”, where this species was collected.

Sexual morph: Ascomata of 400–600 × 400–600 µm, apothecioid, superficial, sessile, circular, flattened, with a thick carbonaceous rim, exposing a black hymenium at the center; exciple, 45–52 µm wide ($\bar{x}$ = 47.5, *n* = 3), composed of two layers, outer layer of 10–15 µm ($\bar{x}$ = 12.5, *n* = 3), pseudoparenchymatous, black, composed of thick-walled cells 5–5 × 5–6 µm wide, inner layer of 30–35 µm ($\bar{x}$ = 33, *n* = 3), textura prismatica, composed of thick-walled cells; subhymenium of 25–40 µm wide ($\bar{x}$ = 30.71, *n* = 3), hyaline to greenish blue, composed of thick-walled cells; hamathecium 2–3 µm wide, thick-walled, filiform to cylindrical, septate, branched, hyaline pseudoparaphyses, rounded at the apex, forming an olive green (27E) to black thick epithecium over the asci when mounted in KOH ($\bar{x}$ = 25, *n* = 3); bitunicate asci, 70–95 × 10 µm, ($\bar{x}$ = 83 × 10, *n* = 10), cylindrical, clavate, apically rounded, generally straight, with a short pedicel, eight-spored, bitunicate; ascospores 16–20 × 4–5 ($\bar{x}$ = 18.1 × 4.3, *n* = 30), subfusiform, three septa, hyaline to greenish. Asexual morph: unknown.

**Habitat**: Gregarious on dead branches of tree ferns in the genus *Cyathea.*

**Taxonomical notes**: This species is distinguished by having ascomata of 400–600 × 400–600 µm and for the size of the ascospores (16–20 × 4–5), which are fusiform and three septa. Morphologically, it is similar to *Patellaria andina*; they have the same number of septa in the ascospores, but they differ by the size of the ascospores ($\bar{x}$ = 18.1 vs. $\bar{x}$ = 22–25 µm). *P. esperanzae* and *P. andina* are not closely related in the phylogenetic analysis.

In the present phylogenetic analysis, *P. esperanzae* is related to *P. mangrovei*. However, P. esperanzae differs from *P. mangrovei* by the epithecium color (olive green (27E) vs. olive brown (4E8)) and by having smaller ascospores (18.1 × 4.3 µm vs. 29.36 × 6.83 µm) and fewer septa (3 vs. 6–8). Additionally, *P. esperanzae* grows on tree ferns of the genus *Cyathea* in tropical montane cloud forest in Oaxaca, while *P. mangrovei* grows on *Rhizophora mangle* in a mangrove community in Quintana Roo.

***Patellaria esquedii*** García-Jacobo, Raymundo, and R. Valenz. sp. nov.

**MycoBank**: 855493.

**Figure 5:**
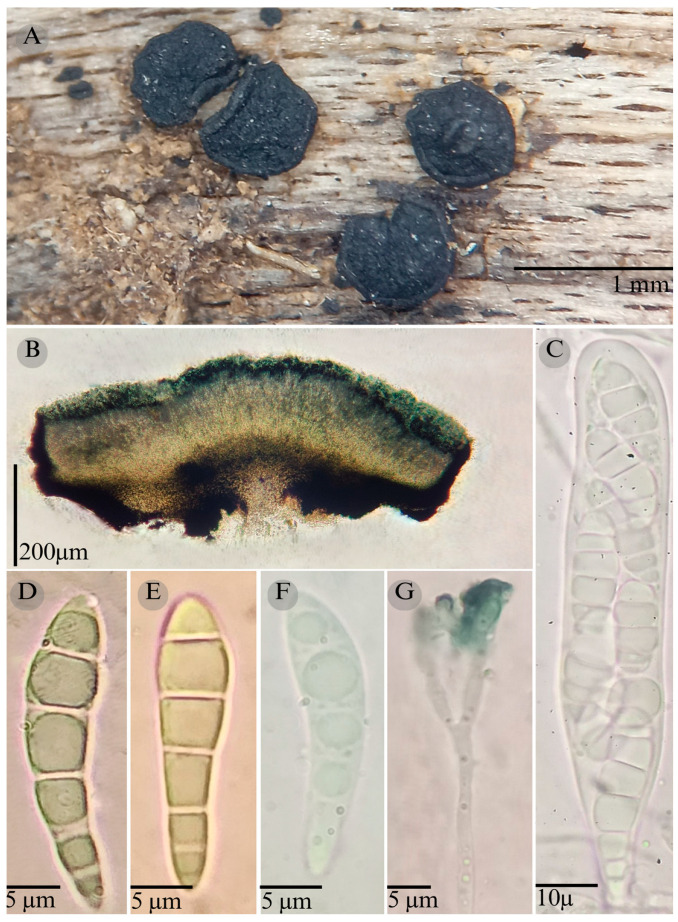
*Patellaria esquedii* García-Jacobo, Raymundo, and R. Valenz. (**A**) Ascomata, (**B**) longitudinal section of ascomata, (**C**) asci, (**D**–**F**) ascospores, and (**G**) pseudoparaphysis.

**Figures:** Figure 1, Figure 2 and Figure 5.

**TYPE**: Mexico. Campeche, Imí municipality, Km. 179 de la Carretera Champotón–Mérida, Kopomá, 20°37′21″ N, 89°56′23″ W, 18 m, 19 January 2018, T. Raymundo 7255 (ENCB, Holotype).

**Etymology**: The epithet honors Dr. Martín Esqueda for his important contributions to Mexican mycology.

Sexual morph: Ascomata of 600–1000 × 400–700 µm, apothecioid, superficial, sessile, circular, flattened, with a thick carbonaceous rim, exposing a black hymenium at the center; peridium 35–40 µm wide ($\bar{x}$ = 37.3, *n* = 10), composed of two layers, outer layer of 20–24 µm ($\bar{x}$ = 12.5, *n* = 10), pseudoparenchymatous, black, composed of thick-walled cells 4–5 × 6 µm wide, inner layer of 11–13 µm ($\bar{x}$ = 12, *n* = 10), textura prismatica, composed of thick-walled cells; subhymenium of 35–40 µm ($\bar{x}$ = 37.5, *n* = 3), hyaline to greenish blue, composed of thick-walled cells; hamathecium 1.5–3.5 µm wide, thick-walled, filiform to cylindrical, septa, branched, hyaline pseudoparaphyses, rounded at the apex, forming a green jungle (25F8) to black thick epithecium over the asci when mounted in KOH ($\bar{x}$ = 22.3, *n* = 10); bitunicate asci, 85–98 × 13–17 µm ($\bar{x}$ = 91 × 13.83, *n* = 10), cylindrical, clavate, apically rounded, generally straight, with a short unilobed pedicel, eight-spored, bitunicate; ascospores (27) 30–32 × 6 µm ($\bar{x}$ = 29.8 × 6, *n* = 30), obclavate, slightly curved, narrowed at the ends, 5–6 septa, hyaline to greenish. Asexual morph: unknown.

**Habitat**: Gregarious on decaying wood in the tropical rain forest.

**Additional specimens**: Campeche, Imí municipality, Km. 179 de la Carretera Champotón–Mérida, Kopomá, 20°37′21″ N, 89°56′23″ W, 18 m, 19 Jan 2018, R. Valenzuela 15715 (ENCB).

**Notes**: *P. esquedii* is characterized by the size of the asci 85–98 × 13–17 (short pedicellate), the size of the ascospores (27–32 × 6), and the number of septa (5–6) in the ascospores and its obclavate shape.

In this study, *P. esquedii* forms a sister taxon to *Patellaria ramona* and *P. mangrovei*, from which it differs in the shape of the ascospores: clavate in *P. ramona* and *P. mangrovei*, and obclavate in *P. esquedii*. Additionally, the ascospores of *P. esquedii* have 5–6 septa, whereas *P. mangrovei* has 5–8 septa and *P. ramona* has 4–6.

***Patellaria garciae*** García-Jacobo, Raymundo, Martínez-González, and R. Valenz. sp. nov.

**MycoBank**: 855489.

**Figure 6:**
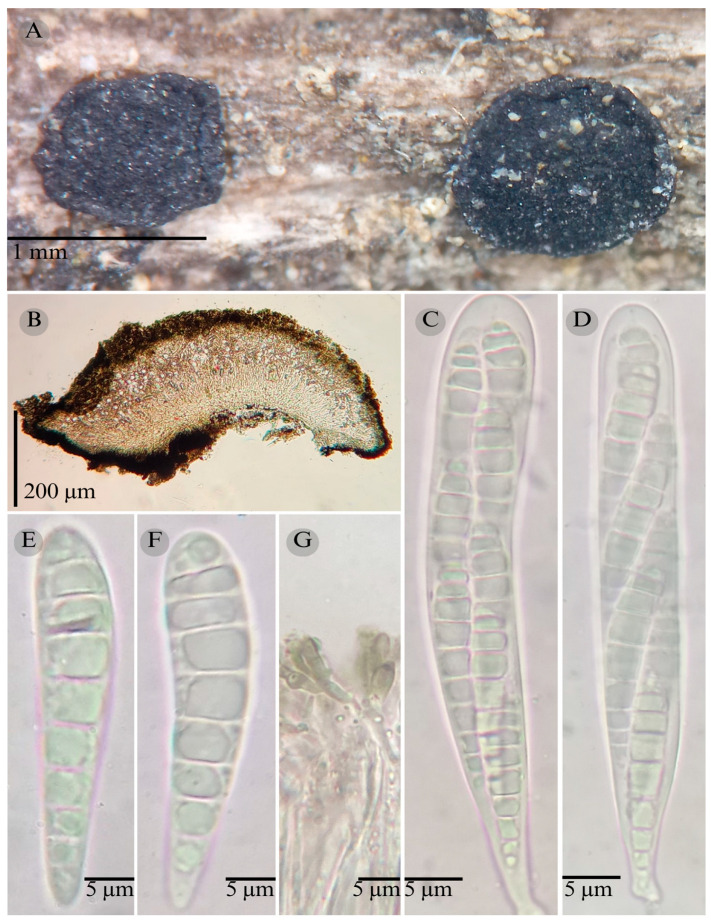
*Patellaria garciae* García-Jacobo, Raymundo, Martínez-González, and R. Valenz. (**A**) Ascomata, (**B**) longitudinal section of ascomata, (**C**,**D**) asci, (**E**,**F**) ascospores, and (**G**) pseudoparaphysis.

**Figures:** Figure 1, Figure 2 and Figure 6.

**TYPE**: Mexico. Coahuila, Arteaga municipality, El Renacer de la Sierra, 25°12′33″ N, 100°22′52″ W, 3177 m, 17 March 2017, T. Raymundo 7425 (ENCB, Holotype).

**Etymology**: In honor of Dr. Jesús García for important contributions to Mexican mycology.

Sexual morph: Ascomata of 700–800 × 700–750 µm, apothecioid, superficial, sessile, circular, flattened with a thin carbonaceous rim, exposing a black hymenium at the center; peridium 50 µm wide ($\bar{x}$ = 5 0 µm, *n* = 3), composed of two layers, outer layer of 25 µm ($\bar{x}$ = 25 µm, *n* = 3), pseudoparenchymatous, black, composed of thick-walled cells 4–6 × 4–6 µm wide, inner layer of 25 µm ($\bar{x}$ = 25 µm, *n* = 3), textura prismatica, composed of thick-walled cells; subhymenium 30 µm (*n* = 3), hyaline to greenish blue, composed of thick-walled cells; hamathecium 1–3 µm wide, thick-walled, filiform to cylindrical, septa, branched, hyaline pseudoparaphyses, rounded at the apex, forming a spruce green (25F3) to black thick epithecium over the asci when mounted in KOH ($\bar{x}$ = 30 µm, *n* = 3); bitunicate asci 100–115 × 15 µm ($\bar{x}$ = 108 × 15 µm, *n* = 10), cylindrical, clavate, apically rounded, generally straight, with a short bilobed pedicel, eight-spored, bitunicate; ascospores, 35–40 (42) × 6–7 (8) µm ($\bar{x}$ = 39.31 × 6.76 µm, *n* = 30), clavate, slightly curved, narrowed at the lower end, 8–9 septa, hyaline refractive in a pale green tone. Asexual morph: unknown.

**Hábitat**: Gregarious on decaying wood in coniferous forests.

**Additional specimens**: Coahuila, Arteaga Municipality, El Renacer de la Sierra, 25°12′33″ N, 100°22′ 52″ W, 3177 m, 17 March 2017, T. Raymundo 7425 (ENCB).

**Notes:** Differs from *P. atrata* by having shorter asci (100–115 × 15 µm), a short pedicel, and smaller ascospores (35–42 × 6–8), with fewer septa (8–9 vs. 5–11).

*Patellaria neoleonensis* is similar to *P. garciae* in the number of septa (8–9 vs. 8–10) but differs by having long pedicellate asci and a dark turquoise epithecium reaction in KOH. These species also differ because *P. neoleonensis* grows in xerophytic scrub and *P. garciae* grows in coniferous forests. Phylogenetically, *P. garciae* is closely related to *P. magenta*, from which it differs morphologically by having smaller asci ($\bar{x}$ = 108 × 15 µm vs. $\bar{x}$ = 120 × 13 µm) and a different ascospore shape: in *P. magenta*, the ascospores are clavate but conic at the apex, while in *P. garciae*, they are clavate and slightly curved. They also differ in the number of septa (8–9 vs. 7–13). Additionally, *P. magenta* grows on unidentified wood in tropical dry forests in Hidalgo, San Luis Potosí, and Sonora, whereas *P. garciae* grows in coniferous forests in Coahuila.

***Patellaria magenta*** García-Jacobo, Raymundo, Mart.-Pineda, and R. Valenz. sp. nov.

**MycoBank**: 855481.

**Figure 7:**
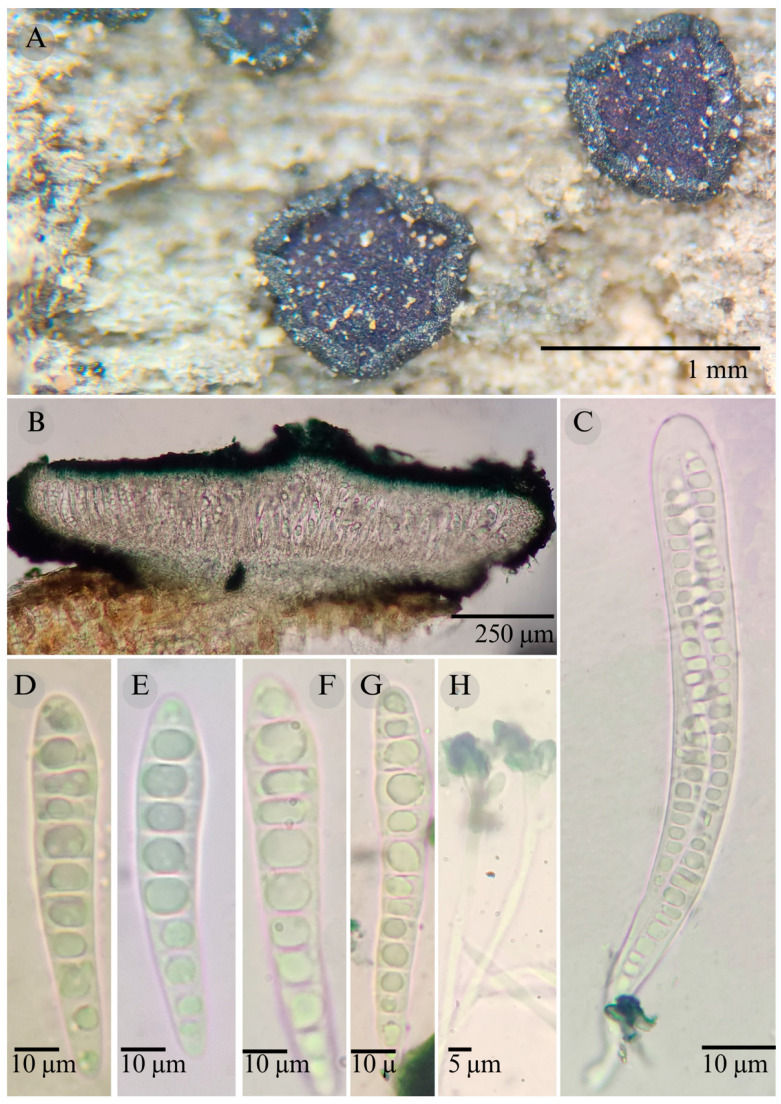
*Patellaria magenta* García-Jacobo, Raymundo, and R. Valenz. (**A**) Ascomata, (**B**) longitudinal section of ascomata, (**C**) asci, (**D**–**G**) ascospores, and (**H**) pseudoparaphysis.

**Figures:** Figure 1, Figure 2 and Figure 7.

**TYPE**: Mexico. Sonora, Álamos municipality, Sierra de Álamos-Río Cuchujaqui Biosphere Reserve, Promontorios, 27°00′54.1″ N, 109°02′10.5″ W, 602 m, 7 October 2013, T. Raymundo 5427 (ENCB, Holotype).

**Etymology**: The epithet refers to the color of the hymenium.

Sexual morph: Ascomata of 600–930 × 660–1000 µm, discoidal, superficial, sessile, circular, flattened, with a thick and involute carbonaceus rim, exposing a black to dark ruby (12F8) hymenium at the center; peridium 37–56 µm wide ($\bar{x}$ = 46.5 µm, *n* = 10), composed of two layers, outer layer 20–27 µm wide ($\bar{x}$ = 23.6 µm, *n* = 10) pseudoparenchymatous, black, composed of thick-walled cells 5–5 × 6 µm wide, inner layer 20–29 µm wide ($\bar{x}$ = 24.25 µm, *n* = 10), textura prismatica, composed of thick-walled cells; subhymenium 25–37 µm wide ($\bar{x}$ = 30 µm, *n* = 30), hyaline to greenish blue, composed of thick-walled cells; hamathecium 1–3 µm wide, thick-walled, cylindrical, septate, branched, hyaline pseudoparaphyses, rounded at the apex, forming a green jungle (25E7) to thick black epithecium over the asci when mounted in KOH; bitunicate asci 95–145 × 12–20 µm, ($\bar{x}$ = 120 × 13 µm, *n* = 10), cylindrical, clavate, apically rounded, generally straight, with a long bilobed pedicel, eight-spored, bitunicate; ascospores (30) 32–37 (54) × 6–8 µm, ($\bar{x}$ = 36 × 6 µm, *n* = 30), clavate, conic at the apex, narrowed at the lower end, (7) 8–10 (13) septa, and hyaline refractive in green tone. Asexual morph: unknown.

**Habitat**: Gregarious on decaying wood in tropical dry forests.

**Additional specimens**: Hidalgo, Tepeapulco municipality, Zona Arqueológica El Xihuingo, 19°48′44″ N, 98°33′12″ W, 2499 m, 9 June 2024, P.M. Alvarez-Cortés 520 (ENCB). San Luis Potosí, Villa Hidalgo municipality, Km. 88 Carretera San Luis– Río Verde, 22°31′11″ N, 100°39′43″ W, 1572 m, 18 June 2023, T. Raymundo 9218 (ENCB). Sonora, Álamos municipality, Sierra de Álamos-Río Cuchujaqui Biosphere Reserve, Promontorios, 27°00′54.1″ N, 109°02′10.5″ W, 602.2 m, 7 October 2014, R. Valenzuela 15818 (ENCB); 26 October 2018, T. Raymundo 8071 (ENCB); R. Valenzuela 18751 (ENCB).

**Notes:** *Patellaria magenta* is characterized by its purple epithecium color and the size (30–54 × 6–8) and number of septa on its ascospores (7–13). *P. neoleonensis* is similar in the ascospore size (32–50 × 6–8) and the long pedicellate asci but differs in the KOH reaction (dark turquoise: 24F8) and has fewer septa (8–10).

*Patellaria magenta* is phylogenetically related to *P. tropicalis* and *P. neoleonensis*, which forms a sister clade with *P. magenta. P. tropicalis* and *P. magenta* can be distinguished by the size of the asci ($\bar{x}$ = 120 × 13 µm vs. $\bar{x}$ = 85.12), the size of the ascospores ($\bar{x}$ = 36 × 6 µm vs. $\bar{x}$ = 28.3 × 6.12 µm), and the number of septa (7–13 vs. 5–7).

***Patellaria mangrovei*** García-Jacobo, M. Mtz.–Pineda, R. Valenz, and Raymundo sp. nov.

**MycoBank**: 855488.

**Figure 8:**
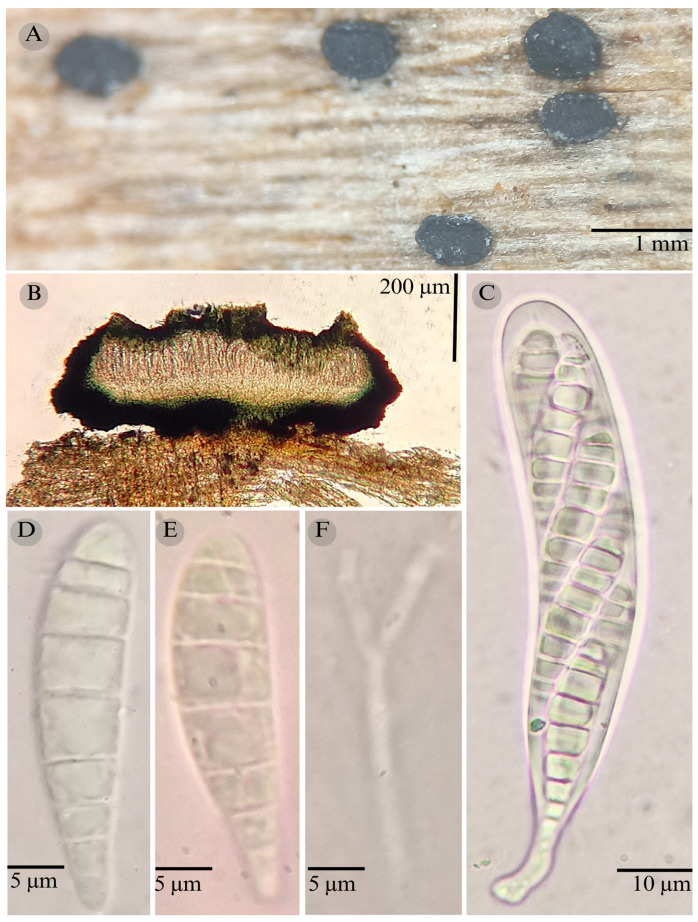
*Patellaria mangrovei* García-Jacobo, M. Mtz.–Pineda, R. Valenz, and Raymundo. (**A**) Ascomata, (**B**) longitudinal section of ascomata, (**C**) asci, (**D**,**E**) ascospores, and (**F**) pseudoparaphysis.

**Figures:** Figure 1, Figure 2 and Figure 8.

**TYPE**: Mexico. Quintana Roo, San Miguel de Cozumel municipality, Parque Ecológico Punta Sur, Cozumel Island Biosphere Reserve, 20°18′01″ N, 87°00′0″ W, 1 m, 15 October 2019, T. Raymundo 8326 (ENCB, Holotype).

Etymology: Name refers to the fact that the species grows on mangroves of genus *Rhizophorae*.

Sexual morph: Ascomata of 500–700 × 500–600 µm, apothecioid, superficial, sessile, circular, flattened, with a thin carbonaceous rim, exposing a black hymenium at the center; peridium 40–43 µm wide ($\bar{x}$ = 42 µm, *n* = 3), composed of two layers, outer layer of 20–22 µm ($\bar{x}$ = 20.5 µm, *n* = 10), pseudoparenchymatous, black, composed of thick-walled cells 4–5 × 4–5 µm wide, inner layer of 20–23 µm ($\bar{x}$ = 22.6 µm, *n* = 10), textura prismatica, composed of thick-walled cells; subhymenium of 60 µm (*n* = 3), hyaline to greenish blue, composed of thick-walled cells; hamathecium 1–3 µm wide, thick-walled, filiform to cylindrical, septate, branched, hyaline pseudoparaphyses, rounded at the apex, forming an olive brown (4E8) to black thick epithecium over the asci when mounted in KOH ($\bar{x}$ = 20, *n* = 3); bitunicate asci 75–90 × 14–16 µm, ($\bar{x}$ = 83 × 15 µm, *n* = 10), cylindrical, clavate, apically rounded, generally straight, short pedicellate, eight-spored, bitunicate; ascospores (25) 29–30 (35) × 6–7 (9) µm, ($\bar{x}$ = 29.36 × 6.83 µm, *n* = 30), clavate, slightly curved, narrowed at the lower end, 5–8 septa, hyaline refractive in a pale green tone. Asexual morph: unknown.

**Habitat**: Gregarious to solitary on decaying wood of *Rhizophora mangle* L.

**Notes**: *Patellaria mangrovei* is distinguished from other *Patellaria* species by the epithecium color in the presence of KOH (olive brown). This species grows on decaying wood of *Rhizophora mangle*. *Patellaria potosina* is similar in that it has 5–8 septate ascospores, but in this species, 8 septa is rare. It differs by the reaction in KOH (nautical blue: 24E7) and the ascospore size (25–35 × 6–9 vs. 31–37 × 5–7), and shape; *P. mangrovei* ascospores are clavate, and those of *P. potosina* are obclavate.

With regard to other mangrove species, *P. apiculatae* has been reported growing on *Rhizophora apiculata.* These species can be distinguished by morphological features, such as the color of the epithecium in KOH (olive brown vs. bluish-black), the size of the asci ($\bar{x}$ = 83 × 15 µm vs. $\bar{x}$ = 62 × 16 µm), with *P. mangrovei* having larger asci, and the number of septa in the ascospores (5–8 vs. 5–6). Furthermore, these species are not closely related in terms of their phylogeny.

***Patellaria neoleonensis*** García-Jacobo, Raymundo and R. Valenz. sp. nov.

**MycoBank**: 855491.

**Figure 9:**
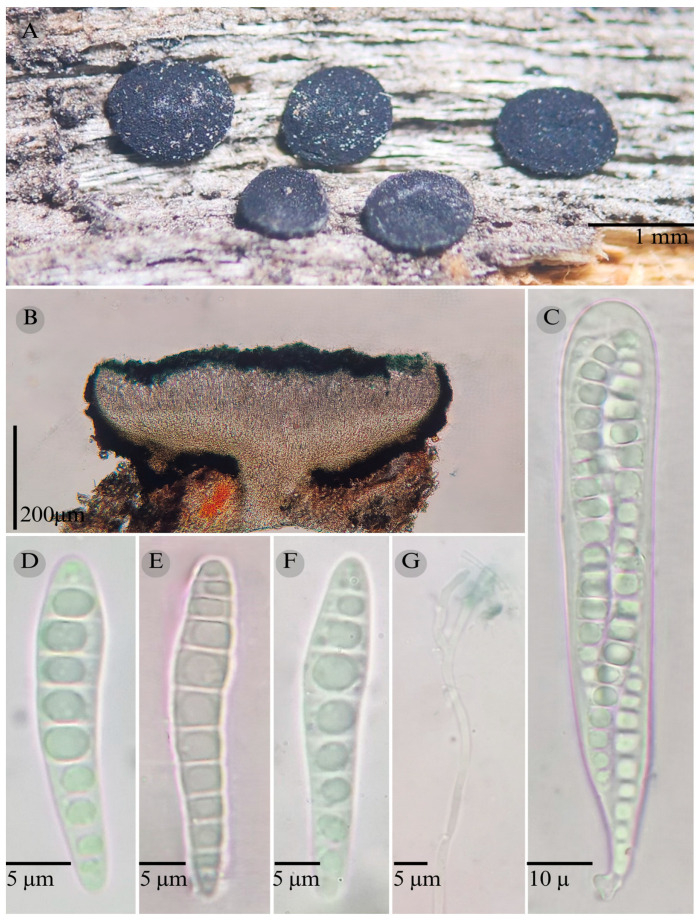
*Patellaria neoleonensis* García-Jacobo, Raymundo, and R. Valenz. (**A**) Ascomata, (**B**) longitudinal section of ascomata, (**C**) asci, (**D**–**F**) ascospores, and (**G**) pseudoparaphysis.

**Figures:** Figure 1, Figure 2 and Figure 9.

**TYPE**: Mexico. Nuevo León, Sabinas Hidalgo municipality, La Cuchilla, 26°29′07″ N, 100°12′52″ W, 338 m, 22 October 2017, R. Valenzuela 17594 (ENCB, Holotype).

**Etymology**: The epithet refers to the state where this species was collected.

Sexual morph: Ascomata of 830–1000 × 700–1000 µm, apothecioid, superficial, sessile, circular, flattened, exposing a black hymenium at the center, with a thick, involute, and striate carbonaceous rim; peridium 45–62 µm wide ($\bar{x}$ = 55 µm, *n* = 10), composed of two layers, outer layer of 25–35 µm ($\bar{x}$ = 28.6 µm, *n* = 10), pseudoparenchymatous, black, composed of thick-walled cells 5–5 × 6 µm wide, inner layer of 20 to 37 µm ($\bar{x}$ = 27.3 µm, *n* = 10), textura prismatica, composed of thick-walled cells; subhymenium 20–35 µm wide ($\bar{x}$ = 28.33 µm, *n* = 3) greenish blue, composed of thick-walled cells; hamathecium 1.5–3.5 µm wide, thick-walled, filiform to cylindrical, septate, branched, hyaline pseudoparaphyses, rounded at the apex, forming a dark turquoise (24F8) to black thick epithecium over the asci when mounted in KOH ($\bar{x}$ = 21, *n* = 10); bitunicate asci 93–154 × 12–20 µm, ($\bar{x}$ = 109.7 × 14.7 µm, *n* = 10), cylindrical, clavate, apically rounded, generally straight, with a short bilobed pedicel, eight-spored; ascospores (32) 38–42 (50) × 6–8 µm, ($\bar{x}$ = 39.87 × 6.6 µm, *n* = 30), clavate, narrowed at the lower end, 8–10 septa, hyaline refractive in a pale green tone. Asexual morph: unknown.

**Habitat**: Gregarious on decaying wood in xerophilous scrub.

Additional studied specimens: Nuevo León, Sabinas Hidalgo municipality, La Cuchilla, 26°29′07″ N, 100°12′52″ W, 338, 22 October 2017, T. Raymundo 7050 (ENCB); R. Valenzuela 17597 (ENCB).

**Notes**: Patellaria neoleonensis can be differentiated by the color of its reaction in KOH (spruce green: 25F3) and grows in xerophilous scrub. *Patellaria garciae* is similar, having clavate ascospores and 8–9 septa but differs by growing in coniferous forests and its KOH reaction (dark turquoise: 24F8). In this work, this species is phylogenetically related to *P. tropicalis*, and they differ morphologically in having larger asci ($\bar{x}$ = 85.12 µm vs. $\bar{x}$ = 109.7 µm), larger ascospores ($\bar{x}$ = 39.87 vs.$\bar{x}$ = 28.3 µm), and more septa (8–10 vs. 5–7).

**Figure 10:**
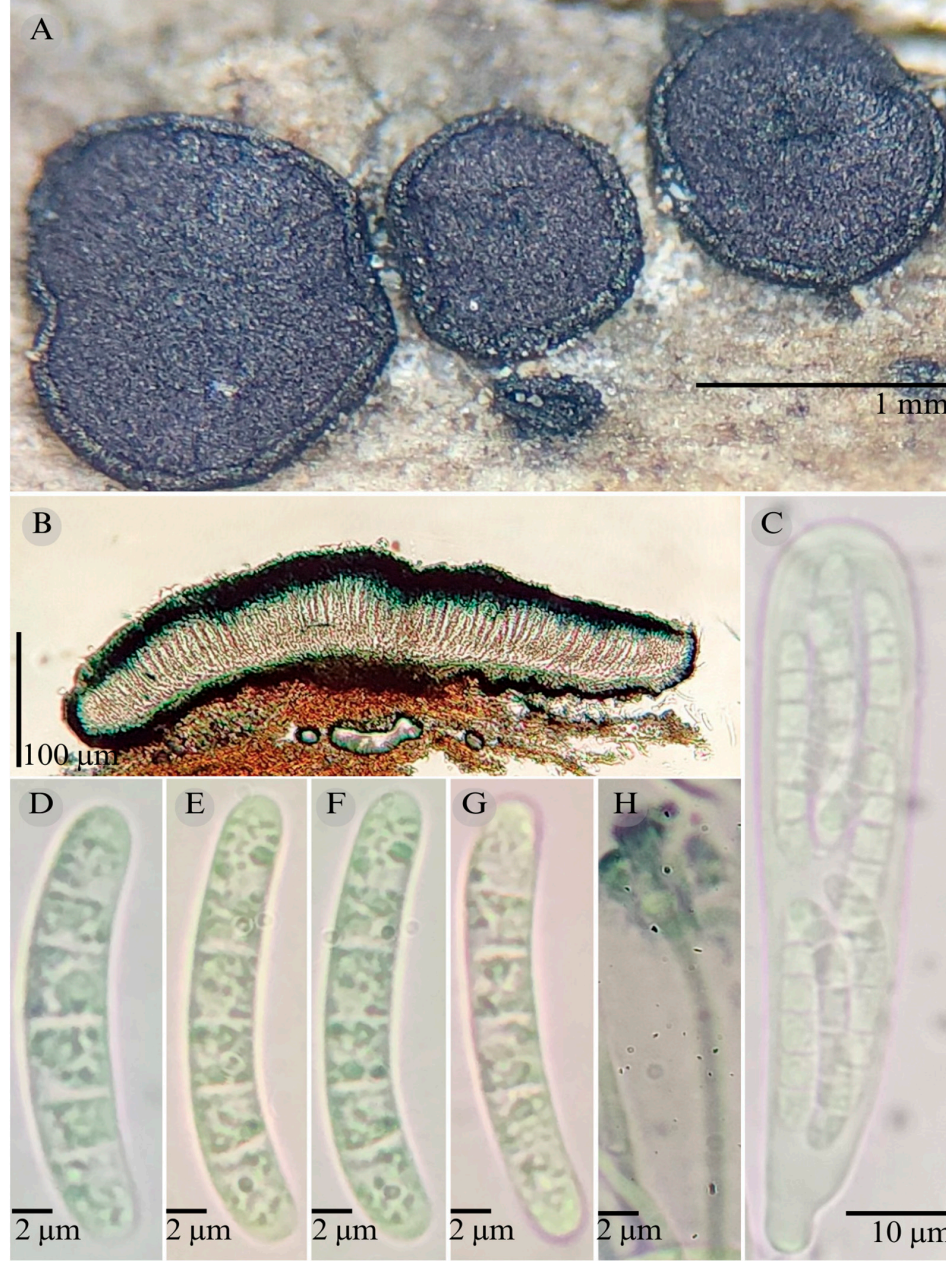
*Patellaria politecnica* García-Jacobo, Raymundo, Mart.-Pineda, and R. Valenz. (**A**) Ascomata, (**B**) longitudinal section of ascomata, (**C**) asci, (**D**–**G**) ascospores, and (**H**) pseudoparaphysis.

***Patellaria politecnica*** García-Jacobo, Raymundo, Mart.-Pineda, and R. Valenz. sp. nov. Figure 1, Figure 2 and Figure 10.

**MycoBank**: 855487.

**Figures:** Figure 1, Figure 2 and Figure 10.

**TYPE**: Mexico. Nayarit. San Blas municipality, Km 27 carretera San Blas–Tepic, 21°33′09″ N, 105°05′32″ W, 577 m, 29 September 2018, T. Raymundo 7979 (ENCB, Holotype).

**Etymology**: This species is dedicated to the Instituto Politécnico Nacional for its support in the study of Dothideomycetes in Mexico.

Sexual morph: Ascomata of 700–860 × 500–800 µm, apothecioid, superficial, sessile, circular, flattened, with a thin carbonaceous rim, exposing a black hymenium at the center; peridium 25–30 µm wide ($\bar{x}$ = 28.3 µm, *n* = 10), composed of two layers, outer layer of 10–17 µm ($\bar{x}$ = 14.33 µm, *n* = 10), pseudoparenchymatous, black, composed of thick-walled cells 4–5–5 × 6 µm wide, inner layer of 12 to 25 µm ($\bar{x}$ = 16.7 µm, *n* = 10), textura prismatica, composed of thick-walled cells; subhymenium of 10 µm (*n* = 3), hyaline to greenish blue, composed of thick-walled cells; hamathecium 1–3 µm wide, thick-walled, filiform to cylindrical, septate, branched, hyaline pseudoparaphyses, rounded at the apex, forming a nautical blue (24E7) to black thick epithecium over the asci when mounted in KOH ($\bar{x}$ = 18.3 µm, *n* = 10); bitunicate asci of 55–76 × 10–15 µm ($\bar{x}$ = 65.18 × 12.60 µm, *n* = 10), cylindrical, clavate, apically rounded, generally straight, with a short unilobed pedicel, eight-spored, bitunicate; ascospores 21–26(27) 32 × 4–5 µm ($\bar{x}$ = 25 × 4.04 µm, *n* = 30), allantoid, (3) 4–6 septa, and hyaline refractive in a pale green tone. Asexual morph: unknown.

**Habitat**: Gregarious on decaying wood in the tropical rain forest.

**Additional specimens**: Campeche, Kopomá municipality, Km. 179 Carretera Champotón–Mérida, 20°37′21″ N, 89°56′23″ W, 18 m, 19 January 2018, T. Raymundo 7256 (ENCB). Nayarit. San Blas municipality, Km. 27 carretera San Blas–Tepic, 21°33′09″ N, 105°05′32″ W, 577 m, 29 September 2018, R. Valenzuela 18679 (ENCB), M. Sánchez 1392 (ENCB).

**Notes**: *Patellaria politecnica* differs from other *Patellaria* species by having a thinner subhymenium (10 µm) and distinctively sized ascospores (21–32 × 4–5 µm). The allantoid ascospores found in *P. chromolaenae* are differentiated from those of *P. politecnica* by the number of septa (4–6 vs. 5–6) and the hymenium color, which is black in *P. chromolaenae* and litmus bluish red in *P. politecnica*. *P. politecnica* is phylogenetically close to *P. andina*, a species described from Argentina that grows on decorticated branches of *Prosopis alpataco. P. politecnica* differs morphologically from *P. andina* by having larger ascomata (700–860 × 500–800 µm vs. 250–500 µm) and differently shaped ascospores (allantoid vs. cylindrical). Thay also differ in the number of septa in the ascospores (4–6 vs. 3).

***Patellaria potosina*** García-Jacobo, Raymundo and R. Valenz. sp. nov.

**MycoBank**: 855482.

**Figure 11:**
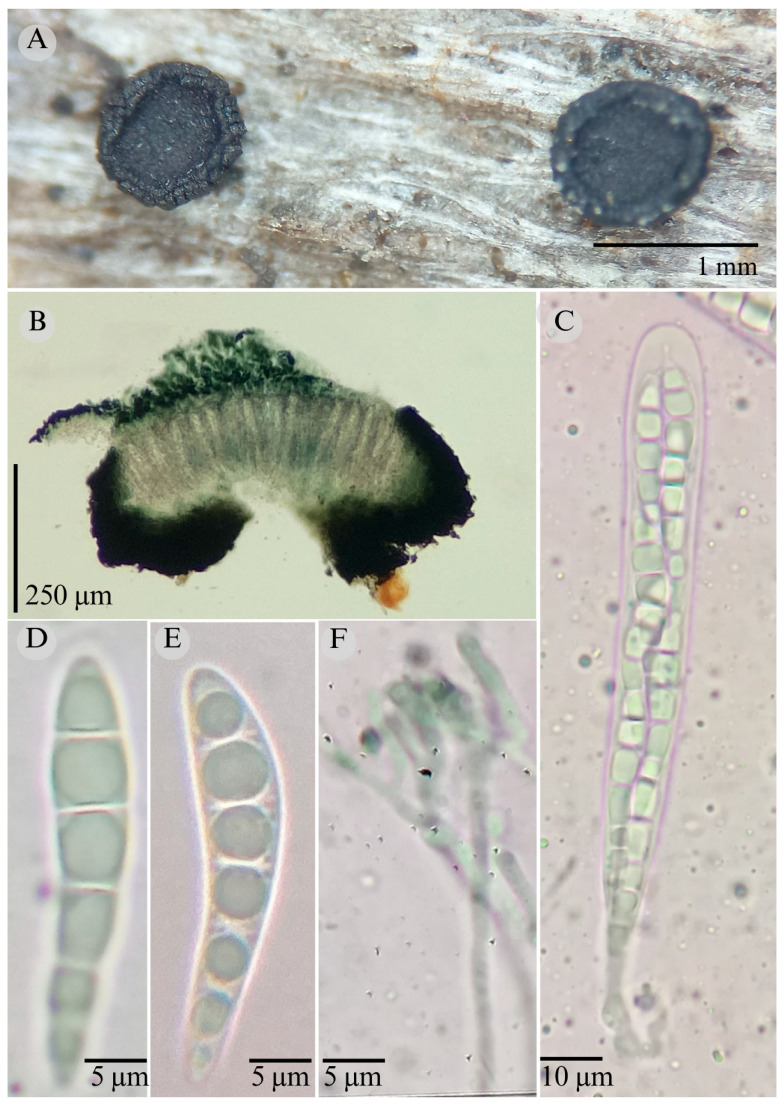
*Patellaria potosina* García-Jacobo, Raymundo, and R. Valenz. (**A**) Ascomata, (**B**) longitudinal section of ascomata, (**C**) asci, (**D**,**E**) ascospores, (**F**) pseudoparaphysis.

**Figures:** Figure 1, Figure 2 and Figure 11.

**TYPE**: Mexico. San Luis Potosí, Rayón municipality, Km. 65 Carretera Cd. Valles–Río Verde, 21°51′51″ N, 99°30′01″ W, 1123 m, 14 September 2015; T. Raymundo 5307 (ENCB, Holotype).

**Etymology**: The epithet refers to the state where the species was collected.

Sexual morph: Ascomata of 550–750 × 550–730 µm, discoidal, superficial, sessile, circular, flattened, with a thin, striate carbonaceus rim, involute, exposing a black hymenium at the center; peridium 35–45 µm wide ($\bar{x}$ = 40 µm, *n* = 10), composed of two layers, outer layer of 15–20 µm ($\bar{x}$ = 18 µm, *n* = 10), pseudoparenchymatous, black, composed of thick-walled cells 3.5–5 × 4–5 µm wide, inner layer of 20 µm ($\bar{x}$ = 20 µm, *n* = 10), textura prismatica, composed of thick-walled cells; subhymenium of 40–55 µm ($\bar{x}$ = 45 µm, *n* = 3), hyaline to greenish blue, composed of thick-walled cells; hamathecium 1.5–3 µm wide, thick-walled, filiform to cylindrical, septate, branched, hyaline pseudoparaphyses forming a nautical blue (24E7) to black thick epithecium over the asci when mounted in KOH ($\bar{x}$ = 22.5, *n* = 10); bitunicate asci 75–116 × 11–15 µm ($\bar{x}$ = 91.42 × 12.56 µm, *n* = 10), a cylindrical, clavate, apically rounded, generally straight appearance, with a long bilobed pedicel, eight-spored, bitunicate; ascospores (30)31–33(38) × 5–7 µm ($\bar{x}$ = 31.48 × 5.98 µm, *n* = 30), obclavate, slightly curved, narrowed at the lower end, 5–6 (8) septa, and hyaline refractive in green tone. Asexual morph: unknown.

**Habitat**: Gregarious on decaying wood in xerophilous scrub.

**Additional specimens**: San Luis Potosí. Rayón municipality, Km. 65 de la Carretera Cd. Valles–Río Verde, 21°51′51″ N, 99°30′01″ W, 1123 m, 14 September 2015; R. Valenzuela 15715 (ENCB).

**Notes**: *P. potosina* can be recognized by asci 75–116 × 11–15, with bulbous pedicel; ascospores (30)31–33(38) × 5–7 µm, clavate, and 5–8 septa.

*Patellaria mangrovei* is similar to *P. potosina* in that it has ascospores and 5–8 septa but differs in its habitat, with *P. mangrovei* growing on *Rhizhophora mangle* and *P. potosina* growing in the Quercus forest, also called “Chaparral”. *P. mangrovei* also differs in its KOH reaction (olive brown) and the ascospore shapes, which are clavate in *P. mangrovei* and obclavate in *P. potosina*. This species shows phylogenetic affinities to *P. barronii* and *P. ramona* and can be distinguished from *P. barronii* by the epithecium color in KOH (nautical blue (24E7) vs. green jungle (25E7)) and the shape of the pedicel in the asci, which is bilobed in *P. potosina* and unilobed in *P. barronii.* They can also be differentiated by the shape and number of septa (5–6 vs. 6–8) of the ascospores, which are obclavate in *P. potosina* and clavate in *P. barronii. P. potosina* differs from *P. ramona* by the pedicel in the asci, which is unilobed in *P. ramona*, and in the size (The figure refers to the map where the distribution of each species is indicated The figure refers to the map where the distribution of each species is indicated ($\bar{x}$ = 31.48 × 5.98 µm vs. $\bar{x}$ = 21.11 × 6 µm), shape, and septa of ascospores (clavate and 4–6 septa in *P. ramona*). Additionally, *P. ramona* grows in the tropical rain forest.

***Patellaria ramona*** García-Jacobo and Raymundo sp. nov.

**MycoBank**: 855492.

**Figure 12:**
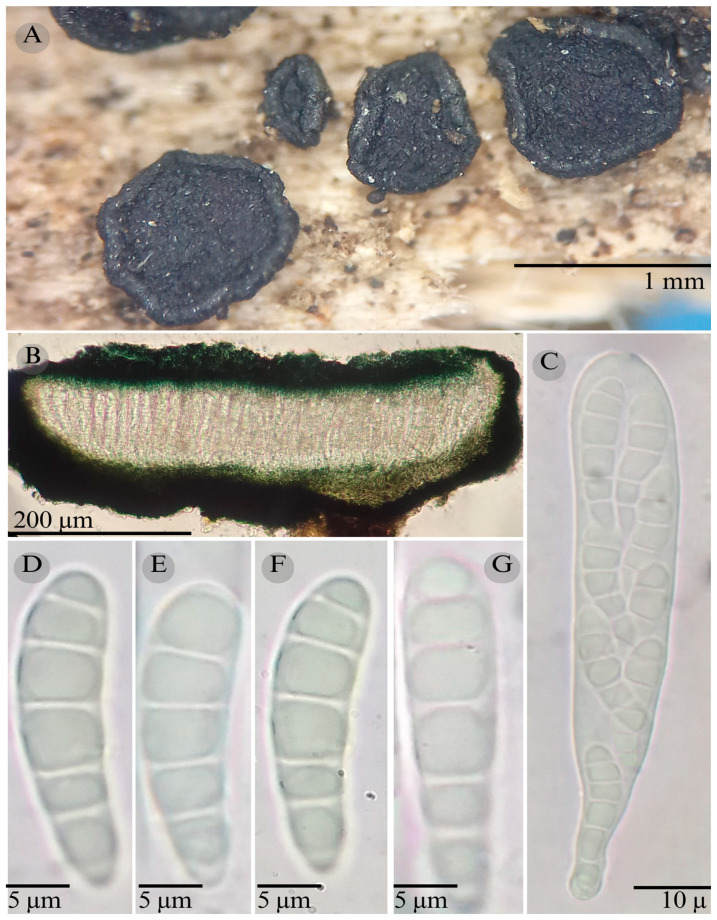
*Patellaria ramona* García-Jacobo and Raymundo. (**A**) Ascomata, (**B**) longitudinal section of ascomata, (**C**) asci, and (**D**–**G**) ascospores.

**Figures:** Figure 1, Figure 2 and Figure 12.

**TYPE**: Mexico. Quintana Roo, Oxtanka archaeological zone, Othón P. Blanco municipality, 18°36′29″ N, 88°14′03″ W, 16 m, 7 February 2021, T. Raymundo 8444 (ENCB, Holotype).

**Etymology**: The epithet refers to the common name of the host where this species grows (Ramón (*Brosimium* sp.)).

Sexual morph: Ascomata of 450–700 × 575–650 µm, apothecioid, superficial, sessile, circular, flattened, with a thin carbonaceous rim, exposing a black hymenium at the center; peridium 25–30 µm wide ($\bar{x}$ = 27.5 µm, *n* = 10), composed of two layers, outer layer of 10–15 µm ($\bar{x}$ = 12.5 µm, *n* = 10), pseudoparenchymatous, black, composed of thick-walled cells 5 × 6 µm wide, inner layer of 15 µm ($\bar{x}$ = 15 µm, *n* = 10), textura prismatica, composed of thick-walled cells; subhymenium of 35–40 µm ($\bar{x}$ = 37.5 µm, *n* = 3), hyaline to greenish blue, composed of thick-walled cells; hamathecium 1.5–3.5 µm wide, thick-walled, filiform to cylindrical, septate, branched, hyaline pseudoparaphyses rounded at the apex, forming a dark turquoise (24F8) to black thick epithecium over the asci when mounted in KOH ($\bar{x}$ = 15, *n* = 3); bitunicate asci 75–96 × 15–17 µm ($\bar{x}$ = 88.5 µm × 15.8, *n* = 10), cylindrical, clavate, apically rounded, generally straight, with a long unilobed pedicel, eight-spored; ascospores (22) 25–29 (30) × 6 µm ($\bar{x}$ = 21.11 × 6 µm, *n* = 30), clavate, slightly curved, narrowed at the lower end, 4–6 septa, hyaline refractive in a pale green tone. Asexual morph: unknown.

**Habitat**: Gregarious on decaying wood in *Brosimium* sp.

**Additional specimens**: Quintana Roo, Othón P. Blanco municipality, Oxtanka archeological zone, “El Ramonal” 18°36′29″ N, 88°14′03″ W, 16 m, 7 February 2021, R. Valenzuela 19244 (ENCB).

**Notes**: *Patellaria ramona* is distinguished by the size of its asci (75–96 × 15–17) with a short pedicell; ascospores are 22–29 × 6, 4–6 septa.

*P. esquedii* is both morphologically and ecologically similar, as it also grows in tropical rain forests. Morphologically, they differ in the color of the epithecium in KOH (dark turquoise (24F8) vs. green jungle (E7)), the length of the asci pedicels (short in *P. ramona* and long in *P. esquedii*), and the shape of the ascospores (clavate in *P. ramona* and obclavate in *P. esquedii*). Another distinguishing feature is the number of septa in the ascospores (4–6 in *P. ramona* and 5–6 in *P. esquedii*).

***Patellaria tropicalis*** García-Jacobo, Raymundo and R. Valenz. sp. nov.

**MycoBank**: 855483.

**Figure 13:**
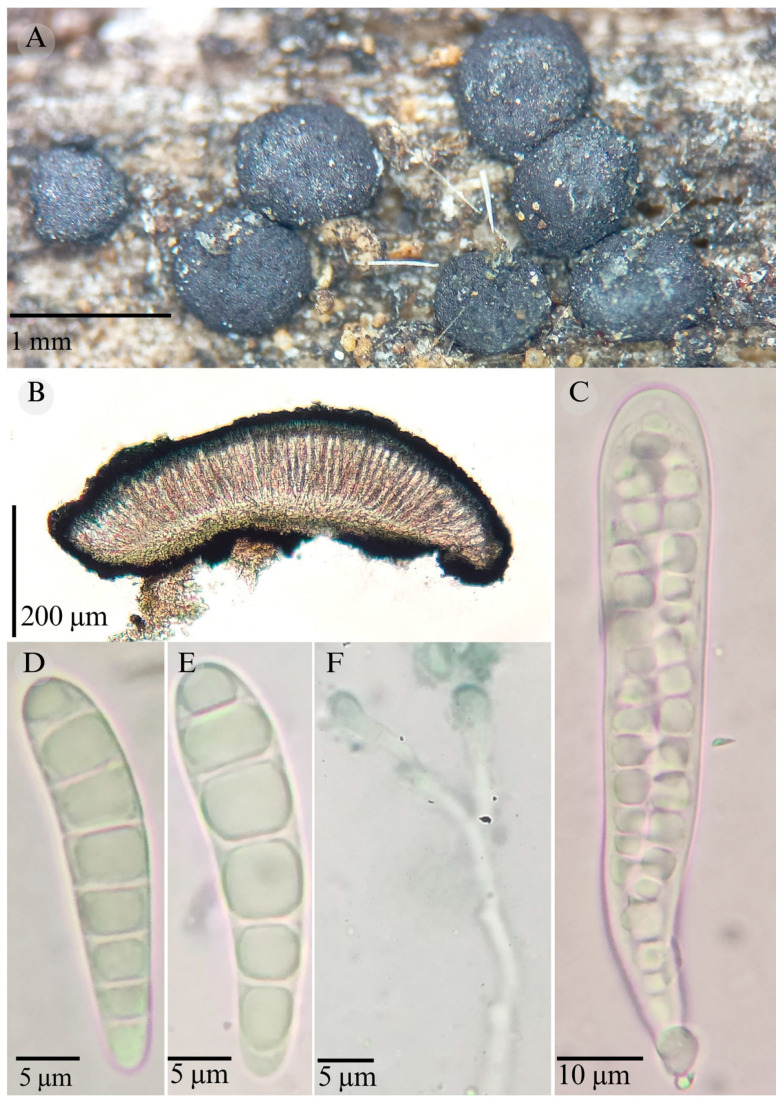
*Patellaria tropicalis* García-Jacobo, Raymundo, and R. Valenz. (**A**) Ascomata, (**B**) longitudinal section of ascomata, (**C**) asci, (**D**,**E**) ascospores, and (**F**) pseudoparaphysis.

**Figures:** Figure 1, Figure 2 and Figure 13.

**TYPE**: Mexico. Sonora, Promontorios, Sierra de Álamos-Río Cuchujaqui Biosphere Reserve 27°00′54.1″ N, 109°02′10.5″ W, 602 m, 3 October 2016, T. Raymundo 8042 (ENCB, Holotype).

**Etymology**: The epithet refers to the vegetation type where the species was collected (tropical dry forest).

Sexual morph: Ascomata of 530–760 × 500–800 µm, discoidal, superficial, sessile, circular, flattened, with a thin carbonaceous rim, exposing a black to dark ruby (12F8) hymenium at the center; peridium 25–40 µm wide ($\bar{x}$ = 39.6 µm, *n* = 10), composed of two layers, outer layer of 15–25 µm ($\bar{x}$ = 18.8 µm, *n* = 10), pseudoparenchymatous, black, composed of thick-walled cells 5–6 × 5–6.5 µm wide, inner layer of 15–40 µm ($\bar{x}$ = 17 µm, *n* = 10), textura prismatica, composed of thick-walled cells; subhymenium of 17 to 30 µm ($\bar{x}$ = 20.28 µm, *n* = 3), hyaline to greenish blue, composed of thick-walled cells; hamathecium 1.5–3 µm wide, thick-walled, filiform to cylindrical, septate, branched, hyaline pseudoparaphyses, rounded at the apex, forming a deep green (25D8) to black thick epithecium over the asci when mounted in KOH ($\bar{x}$ = 20, *n* = 10); bitunicate asci 72–110 × 12–15 µm ($\bar{x}$ = 85.12 × 14.15 µm, *n* = 10), cylindrical, clavate, apically rounded, generally straight, with a unilobed short pedicel, eight-spored, bitunicate; ascospores (24)26–30(37) × 6–7 µm ($\bar{x}$ = 28.3 × 6.12 µm, n = 30), clavate, slightly curved, narrowed at the lower end, 5–7 septa, and hyaline refractive in a pale green tone. Asexual morph: unknown.

**Habitat**: Gregarious on decaying wood in the tropical dry forest.

**Additional specimens**: Sonora, Rancho La Sierrita, Reserva de la Biósfera Sierra de Álamos-Río Cuchujaqui, 28°30′53.2″ N, 111°15′50.5″ W, 528 m, 8 October 2017, T. Raymundo 5486 (ENCB), D. Castro–Bustos 141 (ENCB),; Octubre 27 2018, R. Valenzuela 18730 (ENCB); Promontorios, Sierra de Álamos-Río Cuchujaqui Biosphere Reserve, 27°00′54.1″ N, 109°02′10.5″ W, 602 m, 7 October 2014, T. Raymundo 5404 (ENCB), T. Raymundo 5449 (ENCB); 26 October 2018, T. Raymundo 8070 (ENCB). San Luis Potosí, Sierra del Abra Tanchipa Biosphere Reserve, 22°33′05″ N, 100°29′44″ W, 1522 m, 12 October 2023, R. Valenzuela 19131 (ENCB); 18 December 2023, R. Valenzuela 19250 (ENCB).

**Taxonomical notes:**

Differs from other *Patellaria* species by the size of its asci, 72–110 × 12–15, with short pedicellate; and ascospore size, 24–37 × 6–7 µm, which are clavate, and 5–7 septa.

*P. xerofila* is similar; it has ascospores with 5–7 septa. These species differ in their KOH reaction (dark turquoise vs. deep green) and in the type of vegetation in which they grow; *P. tropicalis* is found in the tropical dry forest and *P. xerofila* in xerophilous scrub. For the species that grow on the same type of vegetation, *P. magenta* is differentiated by its epithecium color (black in *P. tropicalis* vs. dark magenta (13F) in *P. magenta*)), the size of the asci ($\bar{x}$ = 85.12 µm vs. $\bar{x}$ = 120 × 13 µm), and the size ($\bar{x}$ = 28.3 × 6.12 µm vs. $\bar{x}$ = 36 × 6 µm) and shape of the ascospores, which are clavate and conic at the apex in *P. magenta* and clavate in *P. tropicalis.*

However, these species are not closely related phylogenetically.

***Patellaria xerofila*** García-Jacobo, Raymundo, and R. Valenz. sp. nov.

**MycoBank**: 855486.

**Figure 14:**
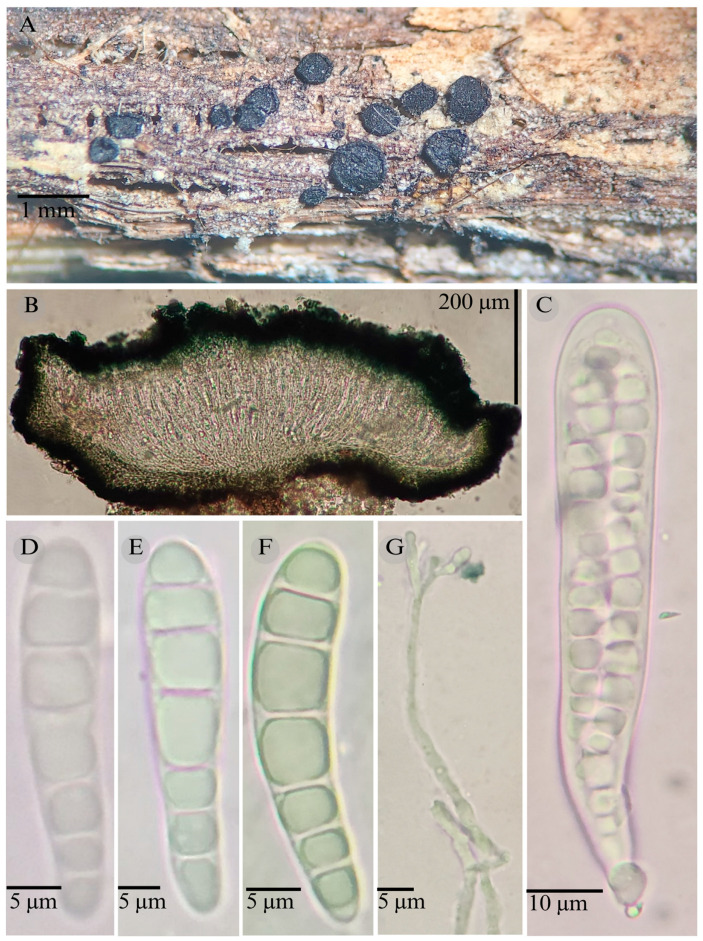
*Patellaria xerofila* García-Jacobo, Raymundo, and R. Valenz. (**A**) Ascomata, (**B**) longitudinal section of ascomata, (**C**) asci, (**D**–**F**) ascospores, and (**G**) pseudoparaphysis.

**Figures:** Figure 1, Figure 2 and Figure 14.

**TYPE**: Mexico. Sonora, San Miguel Horcasitas municipality, 29°29′09″ N, 110°43′03″ W, 397 m, 19 October 2013, R. Valenzuela 15286 (ENCB, Holotype).

**Etymology**: The epithet refers to the type of vegetation where the species grows: xerophilous scrub.

Sexual morph: Ascomata of 510–660 × 510–633 µm, apothecioid, superficial, sessile, circular, flattened, with a thick carbonaceous rim, exposing a black hymenium at the center; peridium 29–45 µm wide ($\bar{x}$ = 36 µm, *n* = 10), composed of two layers, outer layer of 15–30 µm ($\bar{x}$ = 17.5 µm, *n* = 10), pseudoparenchymatous, black, composed of thick-walled cells 4–6.5 × 4–6 µm wide, inner layer of 12 to 25 µm ($\bar{x}$ = 16.7 µm, *n* = 10), textura prismatica, composed of thick-walled cells; subhymenium of 14 to 45 µm ($\bar{x}$ = 25 µm, *n* = 3), hyaline to greenish blue, composed of thick-walled cells; hamathecium 1–3 µm wide, thick-walled, filiform to cylindrical, septate, branched, hyaline pseudoparaphyses, rounded at the apex, forming a dark turquoise (24F8) to black thick epithecium over the asci when mounted in KOH ($\bar{x}$ = 17.5 µm = 10); bitunicate asci 82–110 × 11–15 µm ($\bar{x}$ = 92.9 × 13.03 µm, *n* = 10), cylindrical, clavate, apically rounded, generally straight, short pedicellate, eight-spored; ascospores (26)28–31(39) × 5–7 µm ($\bar{x}$ = 30.9 × 6.02 µm, *n* = 30), clavate, slightly curved, narrowed at the lower end, 5–7 septa, and hyaline refractive in a pale green tone.

**Habitat**: Gregarious on decaying wood in the tropical dry forest and xerophilous scrub.

**Additional specimens**: San Luis Potosí, Rayón municipality, Km. 65 de la Carretera Cd. Valles–Río Verde, 21°51′51″ N, 99°30′01″ W, 1123 m, 14 September 2015, R. Valenzuela 15715 (ENCB). Sonora, San Javier Muncipality, Km. 135 Carretera Hermosillo–Yecora, 28°35′59″ N, 109°48′02″ W, 507 m, 3 October 2016, R. Valenzuela 16965 (ENCB); 16976 (ENCB); 5 October 2016, R. Valenzuela 17124 (ENCB); T. Raymundo 6339 (ENCB); Álamos municipality, El Cajón, Sierra de Álamos-Río Cuchujaqui Biosphere Reserve, 26°51′08″ N, 108°42′24″ W, 355 m, 14 October 2013, T. Raymundo, 4819 (ENCB).

**Taxonomical notes**: *P. xerofila* is distinguished by the size of its asci, 82–110 × 11–15, and the size (26–39 × 5–7) and shape of its ascospores, which are clavate and have 6–7 septa.

*P. tropicalis* is similar in the number of septa but differs mainly in the epithecium reaction in KOH. However, *P. tropicalis* grows in tropical dry forests, and *P. xerofila* grows in xerophilous scrub. Phylogenetically, this species is related to *P. barronii*, *P. ramona*, *P. esquedii*, *P. esperanzae*, and *P. mangrovei*, which forms a sister clade. Species in the sister clade grow in different vegetation types, and only *P. barronii* grows in xerophilous scrub like *P. xerofila*. These species show morphological differences, like the epithecium color in KOH that is dark turquoise (24F8) in *P. xerofila* and green jungle (25E7) in *P. barronii*. They also differ in the number of septa (5–7 vs. 6–8).

 **Key to Patellaria species forming ascomata supported by molecular and morphological data**
1. Species with allantoid ascospores and 3–6 septa ……………………….…………………………………………………………2
1. Species with subfusiform, obclavate, or clavate ascospores; 3–13 septa……………………………………………………………………………3
2. Ascomata < 700 µm in diam; ascospores 27–34 × 5.2–6 µm, allantoid, 3–7 septa; growing in dead stems of Chromolaenae odorata from Thailand …………………………………………………………………*P. chromolaenae*
2. Ascomata > 700 µm in diam; ascospores 21–32 × 4–5 µm, allantoid, 3–6 septa; growing in dead wood of Mexico ……………………………………………………………………*P. politecnica*
3. Ascospores < 20 µm long, subfusiform or claviform …………………………………………………………………………………4
3. Ascospores > 20 µm long, obclavate or claviform …………………………………………………………………………………5
4. Subfusiform ascospores with three septa growing in the stem of the genus Cyathea ……………………………………………………………………*P. esperanzae*
4. Ellipsoid ascospores with eight septa growing in dead wood ……………………………………………………………………*P. microspora*
5. Obclavate ascospores, 5–6 septa …………………………………………………………………………………6
5. Clavate ascospores, 4–13 septa …………………………………………………………………………………7
6. Discoid ascomata, smooth and thick rim, green jungle coloration in KOH (25F8); unilobed pedicellate asci, ascospores 30–32 × 5–7 µm ($\bar{x}$ = 31 × 6, *n* = 30); growing in tropical rain forest ………………………………………………………………………*P. esquedii*
6. Discoid ascomata, striate and thin rim, coloration nautical blue in KOH (24E7); bilobed pedicel asci, ascospores 30–33 × 6–7 µm ($\bar{x}$ = 32 × 6, *n* = 30); growing in Quercus forest “Chaparral” ………………………………………………………………………*P. potosina*
7. Clavate ascospores, slightly curved, 4–8 septa, unilobed asci …………………………………………………………………………………8
7. Clavate ascospores, 8–10 (13) septa, bilobed asci …………………………………………………………………………………15
8. Ascospores < 30 µm long …………………………………………………………………………………9
8. Ascospores > 30 µm long …………………………………………………13
9. Species growing in temperate forests …………………………………………………………………………*P. atrata*
9. Species growing in tropical forests or mangroves…………………………………………………………………10
10. Species growing in mangroves ………………………………………………………………………………11
10. Species growing in tropical forests; ascospores 26–30 µ ………………………………………………………………………………12
11. Ascomata with thick rim and turquoise pigmentation in KOH (24F8), ascospores $\bar{x}$ = 26 × 7, 5–6 septa; growing in *Rhizophora apiculata* ……………………………………………………………………*P. apiculate*
11. Ascomata with a thin rim and olive brown pigmentation in KOH (4E8), ascospores $\bar{x}$ = 29 × 7, *n* = 30, 5–6 septa; growing in *Rhizophora mangle* ……………………………………………………………………*P. mangrovei*
12. Ascomata with thick rim and turquoise pigmentation in KOH (24F8), asci, short pedicellate, ascospores $\bar{x}$ = 28 × 6, *n* = 30, 4–6 septa; growing in tropical rain forests………………………………………………………………*P. ramona*
12. Ascomata with a thin rim with deep green pigmentation in KOH (25D8), asci with long pedicellate, ascospores $\bar{x}$ = 28 × 6, *n* = 30, 5–7 septa; growing in tropical dry forests ………………………………………………………………*P. tropicalis*
13. Ascospores with 5–6 septa ……………………………………………………………*P. ghizhuensis*
13. Ascospores with six or more septa……………………………………………………………………14
14. Ascomata with a thin rim, green jungle pigmentation in KOH (25F8), asci with short pedicellate, ascospores 31–35 × 6–8 µm, $\bar{x}$ = 33 × 7 µm, 6–8 septa; …………………………………………………………*P. barronii*
14. Ascomata with thick rim turquoise pigmentation in KOH (24F8), asci long with pedicellate, ascospores 28–30 × 6–7 µm, $\bar{x}$ = 30 × 6 µm, 5–7 septa…………………………………………………………………*P. xerofila*
15. Species growing in temperate coniferous forest, ascomata with thin and smooth rims, green jungle pigmentation in KOH(CLA), ascospores with clavate blunt apex ……………………………………………………………………*P. garciae*
15. Species in tropical dry forest, ascomata with thick and striate rim, English green pigmentation in KOH, ascospores clavate with conic apex……………………………………………………………………16
16. Ascospores 32–40 × 6–7 µm, $\bar{x}$ = 36 × 6.2 µm, *n* = 30, 8–10 (13) septa, asci with long pedicellate, ……………………………………………………………………*P. purpurea*
16. Ascospores 38–44 × 6–7 µm, $\bar{x}$ = 40 × 6.6 µm, *n* = 30, 8–10 septa, asci with short pedicellate………………………………………*P. neolonensis*

## 4. Discussion

In Mexico, specimens with discoidal, superficial, black ascomata, smaller than 1 mm, green color in KOH, clavate asci, and clavate ascospores with 5–11 septa have been identified as *Patellaria atrata*. Méndez-Mayboca described *Patellaria atrata* growing on sarcocaule shrubland in Puerto Peñasco, Sonora, with ascospores measuring 20–27 × 6–8 µm and 4–5 transverse septa [6]. Chacon and Tapia reported *P. atrata* from Palenque, Chiapas y Perote, Veracruz [5]. However, in recent polyphasic studies, a polyphasic study on fungi has revealed five species described with morphological and molecular data: *P. apiculatae* Dayar. and K.D. Hyde and *P. chromolaenae* Mapook and K.D. Hyde from Thailand [3,11], *P. microspora* Ekanayaka and K.D. Hyde from the United Kingdom [11], and *P. ghizhouensis* J.F. Zhang, Y.Y. Chen, and Z.Y. Liu from China [13]. A detailed morphological revision was made, focusing on taxonomically significant characters such as the peridium, pseudoparaphyses, asci, and ascospores (Table 3) of 37 specimens deposited in a fungal collection at the herbarium of Escuela Nacional de Ciencias Biológicas since 2014. This analysis identified 12 morphospecies, of which none coincided with the description of the type species *P. atrata*. Hence, a phylogenetic analysis was integrated in Figure 1, in which *Patellaria* was recovered as a monophyletic group within Patellariaceae.

In our phylogenetic analysis using three datasets (LSU, SSU, and ITS), Mexican specimens clustered in two clades. The first clade included specimens classified as *Patellaria atrata* from Oregon, USA, phylogenetically related to *P. quercus*, the only *Patellaria* species described as an asexual morph. Only the conidial phase was defined as conidiomata solitary, black, and erumpent, with 1–3 ostiole with brown setae, conidiophores, hyaline, smooth, and slightly pigmented at the base, 1–5 septa, and measuring 10–25 × 3–4 µm [10]. *Patellaria apiculatae* was registered as growing on *Rhizophora apiculata*. The only species in this clade is *P. politecnica*, related to *P. andina* and registered in Argentina. However, *P. politecnica* can be distinguished from alantoid ascospores with 3–6 septa. At the base of this clade are *P. microspora* and *P. guizhouensis*, the first from the UK, and *P. guizhouensis* from Guizhou province in China.

Most Mexican *Patellaria* species are in clade B, where *P. purpurea* is related to *P. garciae*; both have claviform ascospores larger than 40 µm and 8–10 septa. They differ morphologically because *P. purpurea* has ascomata with an epithecium purple and green jungle in KOH and grows in tropical dry forest and xerophilous scrub in Sonoran forest. In contrast, the other species have ascomata with black epithecium and spruce green in KOH and grow in a boreal coniferous forest in the mountain range in Arteaga, Coahuila. These species are related to *P. chroolaenae*. However, these species and *P. politecnica* have alantoid ascospores, which distinguishes them from the other species of the genus. *P. tropicalis* is phylogenetically related to *P. neolenensis*, and both grow in dry climates. However, they differ by having ascomata shorter than 800 µm in diameter. Asci with a long unilobed pedicell, 24–37 × 6–7 µm, and ascospores with 5–7 septa. Ascomata larger than 800 µm diameter, and short pedicellate, bilobed asci, claviform ascospores 32.50 × 6–8 µm, and 8–10 septa, respectively. *P. xerofila* grows in dry climates, too, and it differs by forming a thin and striate peridium, while *P. tropicalis* shows a smooth and thinner peridium. Moreover, the reaction in KOH of *P. xerofila* is dark turquoise (24F8), and in *P. tropicalis*, it is deep green (25D8).

In clade II, *P. mangrovei* is related to *P. esperanzae*, contrasting in morphology and habitat. *P. mangrovei* originates from mangrove communities in Biosphere Reserve Isla Cozumel in the Mexican Caribbean, growing on *Rhizophora mangle* and exhibiting KOH olive brown pigments (4E8) in KOH, with ascospores measuring 29–30 × 6–7 with 5–8 septa. Furthermore, *P. esperanzae* grows on tree ferns of the genus *Cyathea*, and in KOH, its pigments are green olive (27E5), subfusiform ascospores are less than 20 µm, and there are only three septa. *Patellaria esquedii* grows in the tropical rain forests on the coastal plains of the Gulf of Mexico and in the Yucatán Peninsula. It differs from *P. mangrovei* and *P. esperanzae* in ascospore size (25–29 × 6 vs. 31–35 × 5–8 µm) and has fewer septa (4–6 vs. 6–8). This species is closely related to *P. potosina* and differs in the ascospore shape, which is clavate, and in the number of septa (5–6).

## 5. Conclusions

This study provides new insights into the genus in Mexico and emphasizes the key characteristics for identifying Mexican species, some of which are restricted to specific types of vegetation, underscoring the importance of conservation strategies in these areas. This study also provides reference information for the study of the genus in Mexico and other regions of the world.

The most relevant morphological characteristics in the identification of *Patellaria* species are the size of the asci, ascospore shape, and the number of septa, but there are other characters, such as texture and thickness of the peridium and the length and shape of the pedicel, which can be informative in the morphological delimitation of species of the genus.

Additionally, most species are found in xerophilous scrub and tropical dry forests, which shows the affinity of these species for dry climates. This finding provides valuable insights for future studies of the genus in Mexico.

The morphology must not be considered an optional supplement to molecular phylogeny but rather an essential component for species compression and diversification.

## Figures and Tables

**Table 2:** The taxa used in phylogenetic analyses, along with their GenBank accession numbers for nLSU, ITS, and SSU sequence data. “-----” indicates that the sequence was unavailable in GenBank. Accession numbers for sequences generated in this study are denoted in boldface.

Species	Strain No.	GenBank Accession No.		
		nLSU	ITS	SSU
*Glyphium elatum*	EB 0329	KM220937	KM220943	KM220951
*Glyphium elatum*	EB 0342	KM220938	KM220944	KM220952
*Glyphium elatum*	EB 0388	KM220940	KM220946	-----
*Hysteropatella clavispora*	CBS 247.34	AY541493	-----	-----
*Hysteropatella prostii*	H.B. 9934b	KT876980	KT876980	-----
*Hysteropatella prostii*	G.M. 2014-05-20	KM220949	KM220949	-----
*Hysteropatella prostii*	G.M. 2016-02-20.2	MT341324	MT341324	MT341324
*Neohelicosporium acrogenisporum*	MFLUCC 17-2019	MH558871	NR160376	-----
*Neohelicosporium thailandicum*	MFLUCC 16-0221	MF467941	MF467928	MF467928
*Patellaria andina*	G.M. 2000-06-08.222.173-6	MT273251	-----	-----
*Patellaria andina*	G.M. 2003-05-30.716-5	MT293616	MT293616	-----
*Patellaria andina*	G.M. 2003-05-30.715.195-2	MT273250	MT273250	-----
*Patellaria andina*	G.M. 2003-05-28.712-5	MT293619	MT293619	-----
*Patellaria andina*	G.M. 2003-05-28.710-5	MT293624	MT293624	-----
*Patellaria apiculatae*	MFLU 19-1236	MN017860	MN047094	MN017925
*Patellaria atrata*	JAC11495	MK431438	MK432702	MK432702
*Patellaria atrata*	CBS 958.97	GU301855	-----	GU296181
*Patellaria atrata*	G.M. 2013-03-19.1	MN565888	MN565888	-----
*Patellaria atrata*	SQUCC 15117	MW077152	MW077143	-----
*Patellaria barronii*	S. García 1 ENCB	PQ766174	PQ817147	PQ773270
*Patellaria* cf. a*trata*	BCC 28877	GU371829	-----	GU371837
*Patellaria* cf. a*trata*	BCC 28876	GU371828	-----	GU371836
*Patellaria chromolaenae*	MFLUCC 16-0578	MW142387	MW136695	MW127179
*Patellaria chromolaenae*	MFLUCC 17-1482	MT214475	MT214381	MT214426
*Patellaria chromolaenae*	MFLUCC 17-1479	MT214474	MT214380	-----
*Patellaria esperanzae*	T. Raymundo 5700 ENCB	PQ766175	PQ817148	PQ773271
*Patellaria esquedii*	T. Raymundo 7255 ENCB	PQ766176	PQ817149	PQ773272
*Patellaria garciae*	T. Raymundo 7425 ENCB	PQ766177	PQ817150	PQ773273
*Patellaria guizhouensis*	GZCC 19-0222	OR209665	-----	OR134437
*Patellaria mangrovei*	T. Raymundo 8326 ENCB	PQ766178	PQ817151	PQ773274
*Patellaria microspora*	MFLU 16-0567	MW142388	NR175661	
*Patellaria neoleonensis*	R. Valenzuela 17594 ENCB	PQ766179	PQ817152	PQ773275
*Patellaria politecnica*	T. Raymundo 7979 ENCB	PQ766180	PQ817153	PQ773276
*Patellaria potosina*	T. Raymundo 5307 ENCB	PQ766181	PQ817154	PQ773277
*Patellaria magenta*	T. Raymundo 5427 ENCB	PQ766182	PQ817155	PQ773278
*Patellaria magenta*	R. Valenzuela 15818 ENCB	PQ766183	PQ817156	PQ773279
*Patellaria magenta*	T. Raymundo 9218 ENCB	PQ766184	PQ817157	PQ773280
*Patellaria quercus*	CPC 27232	NG059696	NR152540	
*Patellaria ramona*	T. Raymundo 8444 ENCB	PQ766185	PQ817158	PQ773281
*Patellaria tropicalis*	T. Raymundo 8042 ENCB	PQ766186	PQ817159	PQ773282
*Patellaria tropicalis*	R. Valenzuela 19131 ENCB	PQ766187	PQ817160	PQ773283
*Patellaria xerofila*	R. Valenzuela 15286 ENCB	PQ766188	PQ817161	PQ773284

**Table 3:** Characters of *Patellaria* species included in this analysis. XS: Xerophilous scrub, TCMF: Tropical montane cloud forest, TRF: Tropical rain forest, CF: Coniferous forest, TDF: Tropical dry forest, M: Mangrove.

Species	Ascomata	Rim	Exciple	Sub Hymenium	Asci	Pedicel	Epithecium Color & Key	Colour KOH 10%	Ascospores	Septa	Vegetation
*Patellaria barronii*	Discoidal 500–730 µm	Thin striate	33–40 µm $\bar{x}$ = 37	35–40 µm $\bar{x}$ = 37	101.3 × 14.8–15.3 µm ($\bar{x}$ = 101 × 15 µm)	Short unilobed	$\bar{x}$ = 20 µm Color: 25E7 Green Jungle	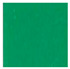	28–36 × 5–8 $\bar{x}$ = 35 × 7 µm Clavate	6–8	XS
*Patellaria esperanzae*	Discoidal 400–600 µm	Thick smooth	45–52 µm $\bar{x}$ = 47.5 µm	25–40 µm $\bar{x}$ = 30 µm	70–95 × 10 µm ($\bar{x}$ = 83 × 10)	-	$\bar{x}$ = 25 µm Color: 27E5 Olive green	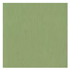	16–20 × 4–5 $\bar{x}$ = 18 × 4 µm Subfusiform	3	TMCF
*Patellaria esquedii*	Discoidal to irregular 600–1000 µm	Thick smooth	35–40 µm $\bar{x}$ = 37.3 µm	35–40 µm $\bar{x}$ = 37 µm	85–98 × 13–17 µm ($\bar{x}$ = 91 × 13.83 µm)	Short unilobed	$\bar{x}$ = 22.3 µm Color: 25E7 Green jungle	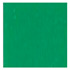	27–32 × 6 $\bar{x}$ = 30 × 6 µm Obclavate	5–6	TRF
*Patellaria garciae*	Discoidal 700–800 µm	Thin smooth	50 µm	30 µm	100–115 × 15 µm ($\bar{x}$ = 108 × 15 µm)	Short bilobuled	$\bar{x}$ = 30 µm Color: 25F3 Spruce green	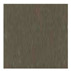	35–42 × 68 $\bar{x}$ = 40 × 7 µm Clavate	8–10	CF
*Patellaria magenta*	Discoidal 600–1000 µm	Thick inovulte striate	40–56 µm $\bar{x}$ = 49.6 µm	25–37 µm $\bar{x}$ = 30.5 µm	95–145 × 12–20 µm ($\bar{x}$ = 120.6 × 13.1 µm)	Long bilobed	$\bar{x}$ = 25 µm Color: 25E7 Green jungle	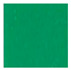	30–54 × 6–8 µm $\bar{x}$ = 36 × 6 Clavate	7–13	TDF, XS
*Patellaria mangrovei*	Discoidal to ovoid 500–700 µm	Thin smooth	40–43 µm $\bar{x}$ = 42	60 µm	75–90 × 14–16 µm ($\bar{x}$ = 83 × 15 µm)	Short unilobed	$\bar{x}$ = 20 µm Color: 4E8 Olive brown	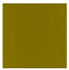	25–35 × 6–9 $\bar{x}$ = 30 × 7 µm Clavate	5–8	M
*Patellaria neoleonensis*	Discoidal 830–1000 µm	Thick involute striate	45–62 µm $\bar{x}$ = 55 µm	20–35 µm $\bar{x}$ = 28	93–154 × 12–20 ($\bar{x}$ = 109.7 × 14.7 µm)	Long bilobed	$\bar{x}$ = 21 µm Color: 24F8 Dark turquoise	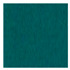	32–50 × 6–8 $\bar{x}$ = 40 × 6 µm Clavate	8–10	XS
*Patellaria politecnica*	Discoidal 700–860 µm	Thin smooth	25–30 µm $\bar{x}$ = 28.3	10 µm	55–76 × 10–15 µm ($\bar{x}$ = 65.18 × 12.60 µm)	Short unilobed	$\bar{x}$ = 18 µm Color: 24E7 Nautic bue	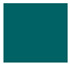	21–32 × 4–5, $\bar{x}$ = 25 × 4 µm Allantoid	3–6	TRF
*Patellaria potosina*	Discoidal 550–750 µm	Thin striate	35–45 µm $\bar{x}$ = 40 µm	40–55 µm $\bar{x}$ = 45 µm	75–116 × 11–15 µm ($\bar{x}$ = 91.42 × 12.56 µm)	Short bilobed	$\bar{x}$ = 22.5 µm Color: 24E7 Nautic bue	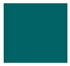	31–38 × 5–7 $\bar{x}$ = 31 × 6 µm Obclavate	5–8	XS
*Patellaria ramona*	Irregular 450–700 µm	Thin smooth	25–30 µm	35–40 µm $\bar{x}$ = 37.5 µm	75–96 × 15–17 µm ($\bar{x}$ = 88.5 × 15.8 µm)	Long bilobed	$\bar{x}$ = 15 µm Color: 24F8 Dark turquoise	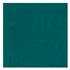	22–29 × 6 $\bar{x}$ = 2 × 6 µm Clavate	4–6	TRF
*Patellaria tropicalis*	Discoidal irregular 530–800 µm	Thin smooth	25–40 µm $\bar{x}$ = 39.6	17–30 µm $\bar{x}$ = 20.28	72–110 × 12–15 µm ($\bar{x}$ = 85.12 × 14.15 µm)	Short unilobed	$\bar{x}$ = 20 µm Color: 25D8 Deep green	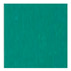	Obclavate 24–37 × 6–7 $\bar{x}$ = 28 × 6 µm Clavate	5–7	TDF
*Patellaria xerófila*	Discoidal irregular 510–660 µm	Thin striate	14–45 µm $\bar{x}$ = 36	14–45 µm $\bar{x}$ = 25	82–110 × 11–15 ($\bar{x}$ = 92.9 × 13.03 µm)	Short unilobed	$\bar{x}$ = 17 µm Color: 24F8 Dark turquoise	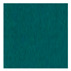	26–39 × 5–7 $\bar{x}$ = 30 × 6 µm Clavate	5–7	XS

## Data Availability

The original contributions presented in the study are included in the article, further inquiries can be directed to the corresponding authors.
